# AHGA-SA: A Novel Adaptive Hybrid Framework for Feature Selection in IoT-Oriented Intrusion Detection with Explainable AI

**DOI:** 10.3390/s26134247

**Published:** 2026-07-04

**Authors:** Saud Abdullah Alzughaibi, Iftikhar Ahmad, Madini Alassafi

**Affiliations:** Department of Information Technology, Faculty of Computing and Information Technology, King Abdulaziz University, Jeddah 21589, Saudi Arabia; iakhan@kau.edu.sa (I.A.); malasafi@kau.edu.sa (M.A.)

**Keywords:** Internet of Things, intrusion detection system, feature selection, genetic algorithm, simulated annealing, adaptive hybrid optimization, explainable AI, Internet of Medical Things, Industrial Internet of Things

## Abstract

The increasing connectivity of Internet of Things (IoT)-oriented environments has made them more vulnerable to cyberattacks, requiring intrusion-detection systems (IDSs) to ensure their secure and reliable operation. The feature selection (FS) process of an IDS affects its performance, as effective FS can enhance detection accuracy and reduce the computational cost and model complexity. This paper presents Adaptive Hybrid Genetic Algorithm-Simulated Annealing (AHGA-SA) as an FS framework that integrates the global search ability of a genetic algorithm and the local exploitation ability of simulated annealing. AHGA-SA aims to find compact, informative feature subsets in high-dimensional intrusion-detection datasets at an acceptable computational cost while maintaining detection performance. The experimental results on three recent benchmarks demonstrate feature-space reduction, with classification accuracies of 99.04% on IoTID20 (using 12 features), 98.25% on WUSTL-EHMS (using seven features), and 99.18% on Edge-IIoTset (using nine features). The results also demonstrate reduced training and testing times, central processing unit usage, resident set size overhead, and subset size compared to the baseline. Furthermore, Shapley additive explanations, as an explainable artificial intelligence technique, are applied to explain the model’s predictions and to show the contribution of the selected features to the IDS decision-making process.

## 1. Introduction

The Internet of Things (IoT) has increased connectivity between devices and systems in many areas, such as healthcare, manufacturing, agriculture, and smart cities [1]. In the medical field, the Internet of Medical Things (IoMT) connects medical devices and healthcare systems to enable patient monitoring and care delivery [2]. In industrial environments, this growth has led to the emergence of the Industrial Internet of Things (IIoT), which enables automation, monitoring, and machine-to-machine communication [3]. While these environments provide valuable operational advantages, their high connectivity and heterogeneity also make them vulnerable to cyberattacks. Attacks such as replay attacks, unauthorized access, and data tampering can affect confidentiality, integrity, and reliability. As a result, effective intrusion-detection systems (IDSs) are critical for ensuring secure and reliable operation in these environments [4,5].

An IDS is a system that identifies malicious activities or potential threats in a network and raises alerts to facilitate timely response. An IDS should be able to detect a variety of attacks with low false positive rate (FPR) and false negative rate (FNR). However, the efficient handling of high-dimensional and diverse intrusion-detection data remains a challenge in IoT-oriented systems [6]. A potential solution to enhance IDS efficiency is feature selection (FS), which selects the most informative features from a set of variables. FS can reduce data dimensionality, leading to better model performance, reduced computational cost, and improved interpretability [7]. Related concerns regarding effective feature representation and vulnerability-aware analysis have also been discussed in adjacent intelligent-system domains [8,9].

FS approaches are typically classified into four categories: filter, wrapper, embedded, and hybrid [10]. Filter methods assess feature relevance using statistical measures. While they are fast and straightforward, they may include redundant or noisy features and may fail to capture feature interactions. Wrapper methods assess feature subsets using learning algorithms and often achieve better detection performance, but they are generally more computationally intensive and prone to overfitting. Embedded methods conduct FS while training models, offering a balance between efficiency and performance, but they are model-dependent. Hybrid methods leverage the strengths of the above methods to perform more effective FS, but they may be harder to design and maintain in large-scale systems. Despite the strengths of these approaches, current FS methods still struggle to simultaneously achieve subset compactness, detection performance, efficiency, and interpretability in diverse IoT-oriented intrusion-detection environments [10,11,12]. Additionally, the balance between exploration and exploitation in high-dimensional search spaces is difficult to achieve in metaheuristic FS, which may increase the risk of convergence to local optima [12,13].

To address these challenges, we present an Adaptive Hybrid Genetic Algorithm-Simulated Annealing (AHGA-SA) method, an FS framework for IDSs in IoT-oriented settings. AHGA-SA integrates the global search ability of a genetic algorithm (GA) and the local exploitation ability of simulated annealing (SA) in a hybrid design that incorporates complementary processes for the discovery of effective feature subsets. In summary, AHGA-SA aims to discover compact and informative feature subsets, maintain or improve detection performance, and enable effective and computationally efficient IDS operation in resource-limited IoT-oriented environments. Finally, explainable artificial intelligence (XAI) is used in the form of Shapley additive explanations (SHAP) to aid in the interpretation of feature contributions and the IDS decision process.

The following are the main contributions of this study:

- We propose AHGA-SA, a novel adaptive hybrid FS framework for IDSs in IoT-oriented environments. The framework integrates GA-based exploration, SA-based exploitation, and Extreme Gradient Boosting (XGBoost)-based feature preselection within a structured two-stage search design for identifying compact and effective feature subsets.
- We evaluate the proposed framework on three recent benchmark datasets representing important IoT-oriented environments, including IoMT and IIoT IDS settings.
- We conduct a comprehensive evaluation using detection performance, computational efficiency, and subset compactness metrics.
- We employ SHAP as an XAI method to interpret the contribution of selected features to the IDS decision process.

The remainder of this paper is organized as follows: Section 2 provides the background and methodological context for this study. Related work is reviewed in Section 3. Section 4 presents the methodology. Section 5 reports the results. Section 6 presents the XAI-based interpretations. Section 7 discusses the findings. Finally, Section 8 concludes this paper and outlines future work.

## 2. Background

This section offers background and context to help understand the proposed framework by introducing the key concepts and methodologies relevant to this study.

### 2.1. FS

FS is a critical step in contemporary machine learning (ML), particularly for high-dimensional data. Such data may lead to redundancy, irrelevant information, and overfitting, which can reduce model performance due to the curse of dimensionality. FS helps overcome these challenges by selecting the most informative subset of features, which can lead to better model performance, lower computational cost, and increased interpretability [7,10].

**Figure 1:**
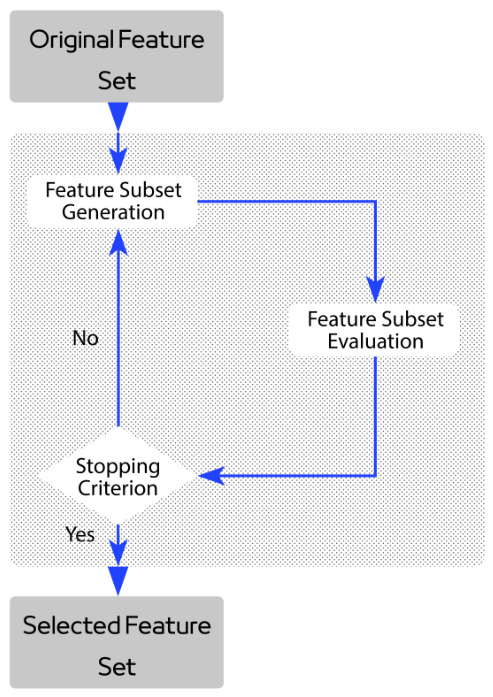
General FS workflow for classification.

FS techniques can be classified into supervised, unsupervised, and semi-supervised methods, depending on the availability of labels. Supervised FS is often used in classification problems because it uses labels to enhance classification performance. The FS process typically consists of four components: subset generation, subset evaluation, the stopping criterion, and the resulting selected feature set [12,14,15]. The general FS process for classification is illustrated in Figure 1.

**Figure 2:**
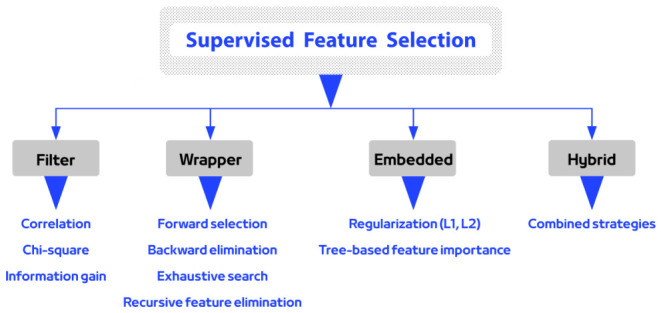
Categorization of supervised FS methods and typical techniques.

In supervised classification, FS techniques are typically classified into four broad categories: filter, wrapper, embedded, and hybrid methods. Figure 2 illustrates these categories and some of their representative techniques. Hybrid approaches combine several FS techniques, such as a filtering step followed by subset refinement [12,13].

**Figure 3:**
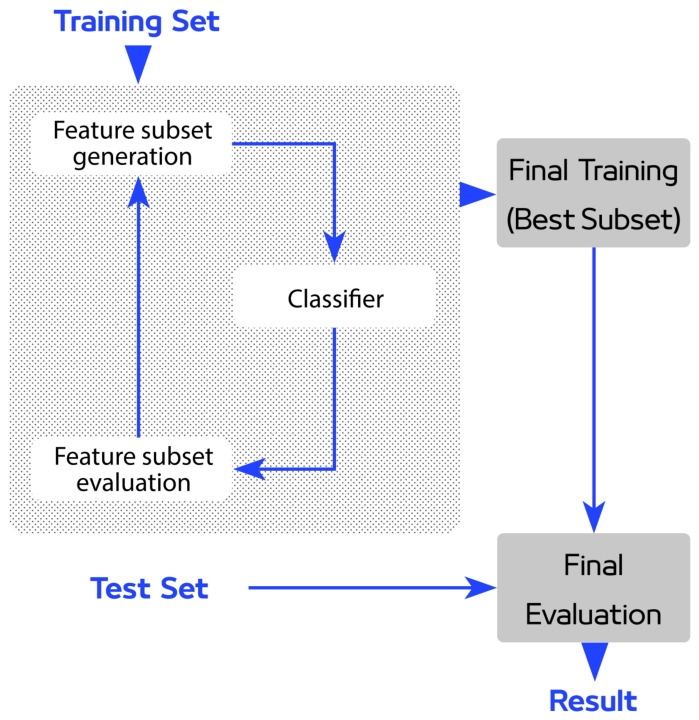
General Framework of wrapper-based FS.

Of these, wrapper-based methods are particularly relevant to optimization-focused FS, as they assess feature subsets based on classification performance, taking into account feature interactions. As illustrated in Figure 3, the wrapper approach iteratively generates and evaluates feature subsets using a classification algorithm to identify the best-performing subset. Wrapper-based methods can employ various search strategies to explore the search space, such as exponential, randomized, and sequential search [16,17]. These strategies can be summarized as follows:

- Exponential search: Searches subsets whose number grows exponentially with the number of features. Therefore, it is often not feasible for high-dimensional data due to its high computational complexity.
- Randomized search: Uses randomness to reduce the risk of converging to local optima and is often population-based, as in many metaheuristic algorithms (MAs).
- Sequential search: Adds or removes features one at a time, as in greedy search, which is fast but may converge to local optima.

### 2.2. MAs

MAs are commonly used to solve complex optimization problems and find optimal or near-optimal solutions. They are typically stochastic and may avoid local optima. MAs do not rely on gradient information and, instead, explore the search space heuristically. MAs can be classified in several ways. They are often divided into two broad categories: trajectory-based and population-based algorithms [18,19].

- Trajectory-based (single-solution) methods: They iteratively improve a single candidate solution, as seen in SA, tabu search, and hill climbing.
- Population-based methods: They work with a population of candidate solutions that are iteratively updated, as in GAs, ant colony optimization, and particle swarm optimization (PSO).

Besides this classification based on the search structure, MAs can also be classified according to their source of inspiration. These include evolution-based (such as GAs), physics-based (such as SA), and swarm-intelligence-based (such as PSO) algorithms. Other classes include chemistry-based, human-inspired, and miscellaneous algorithms [19,20]. Population-based methods are often preferred because their search for multiple solutions can improve exploration and help achieve exploration–exploitation balance [21]. In this study, GAs and SA are of particular interest because the proposed approach uses them as two complementary search components.

### 2.3. Hybrid MAs

Hybrid metaheuristics have attracted considerable attention in optimization research. They can be broadly grouped in three ways: hybridization level, execution model, and control strategy. In terms of the level of hybridization, hybrid approaches can be classified into high-level (weak coupling) and low-level (strong coupling) hybrids. In high-level hybrids, the integrated algorithms interact with each other through clearly defined interfaces but are otherwise independent. Low-level hybrids share internal components or operators in a more tightly integrated manner [22].

In terms of execution, hybrid metaheuristics can adopt batch, interleaved, or parallel approaches, where the hybridized algorithms are executed in a serial, alternate, or parallel fashion. Finally, depending on the control strategy, they may be classified as integrative (coercive), where one algorithm is embedded within another, or cooperative (collaborative), where the algorithms exchange information without direct embedding [23]. Hybridization becomes particularly useful when the strengths of one metaheuristic can compensate for the limitations of another.

### 2.4. GA

**Figure 4:**
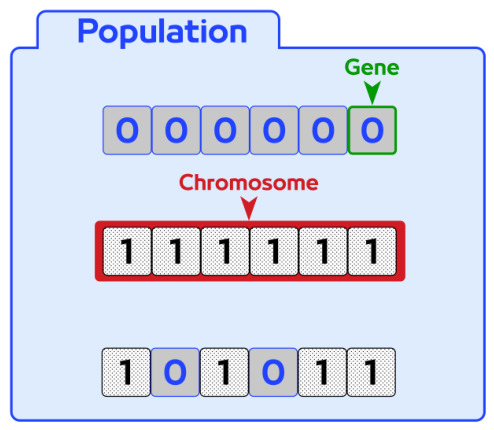
Genes, chromosomes, and population in a GA.

The GA was first introduced by Holland in 1975 as an evolutionary metaheuristic [24]. The key elements of a GA are solution representation (chromosomes), fitness function, selection, and genetic operators (crossover and mutation). The fitness function is used to assess the quality of individuals, and the fitter individuals are given a greater chance of reproduction, according to the principle of natural selection. This process repeats until the termination criteria are met [25]. The relationship between genes, chromosomes, and the population is shown in Figure 4.

Common representation schemes in a GA include the following [26]:

- Binary GA: Represents genes as binary strings (zero or one).
- Real-coded GA: Uses real numbers within bounded ranges and is more appropriate for continuous optimization problems.

**Figure 5:**
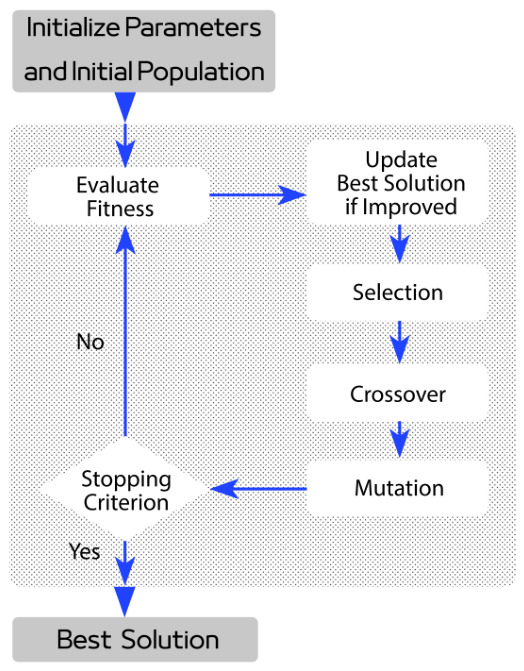
Flowchart of a typical GA.

For discrete problems, the number of bits needed to encode *n* values is $\left\lceil \log_{2}n\right\rceil$ [25]. For FS problems, a binary GA is particularly suitable, as each gene can represent the presence or absence of a feature. The flowchart of a typical GA is shown in Figure 5, and the pseudocode is shown in Algorithm 1.
**Algorithm 1** Typical GA.     **Input:** Population size *N*, crossover rate $C_{r}$, mutation rate $M_{r}$, maximum generations $G_{max}$     **Output:** Best solution $S_{\mathrm{best}}$  1:Initialize population *P* with *N* random solutions  2:Initialize generation counter G←0  3:Evaluate fitness of all individuals in *P*  4:Select the best current individual $x_{\mathrm{best}}$  5:Update $S_{\mathrm{best}}\leftarrow x_{\mathrm{best}}$  6:**while** $G<G_{max}$ **do**  7:      Initialize offspring←[]  8:      **while** |offspring|<N **do**  9:            Select {parent1,parent2} for crossover10:            Apply crossover with rate $C_{r}$ to generate {child1,child2}; otherwise copy the parents11:            Apply mutation (parameterized by $M_{r}$) to {child1,child2}12:            Append {child1} to offspring13:            **if** |offspring|<N **then**14:                 Append {child2} to offspring15:            **end if**16:      **end while**17:      Replace *P* with offspring18:      Evaluate fitness of all individuals in *P*19:      Select the best current individual $x_{\mathrm{best}}$20:      **if** $f\left(x_{\mathrm{best}}\right)>f\left(S_{\mathrm{best}}\right)$ **then**21:            Update $S_{\mathrm{best}}\leftarrow x_{\mathrm{best}}$22:      **end if**23:      Increment generation counter: G←G+124:**end while**25:**return** $S_{\mathrm{best}}$

#### 2.4.1. Initial Population

Initialization of the population is an important phase in a GA, as the quality and diversity of the initial population affect the exploration ability and convergence properties of the algorithm. The key features of initialization methods are as follows [27]:

- Randomness: Indicates how initial solutions are generated, ranging from stochastic approaches to more deterministic or low-discrepancy strategies.
- Compositionality: Indicates the way the initial population is formed, which can be noncomposite or composite (e.g., hybrid or multistep).
- Generality: Refers to whether the initialization method is general or problem-specific.

These characteristics impact the diversity and quality of the initial population, which in turn affects the search process.

#### 2.4.2. Selection

Selection is a core GA process in which individuals are selected for reproduction based on their fitness. Selection is biased toward better solutions, so fitter individuals are more likely to survive and reproduce, which enhances exploitation. At the same time, selection should maintain diversity by giving less-fit individuals a small chance to reproduce, which helps reduce the risk of premature convergence [28]. Common selection strategies include:

- Proportionate roulette wheel selection: Individuals are selected with a probability proportional to their fitness.
- Linear ranking selection: Individuals are selected based on their rank, rather than absolute fitness, which helps reduce the dominance of high-fitness individuals.
- Tournament selection: A random sample of individuals is selected, and the fittest individual is chosen.

Other selection methods, such as exponential ranking, stochastic universal sampling, and truncation, can also be applied depending on the application [29].

#### 2.4.3. Crossover

Crossover creates new offspring by recombining genetic material from parents. This process promotes diversity and enables the search to explore new areas of the search space. It is governed by the crossover rate ($C_{r}$). Higher $C_{r}$ values promote exploration, while lower values help maintain useful schemata and promote exploitation [30]. The following are some common crossover techniques:

- Single-point crossover: One split point is chosen, and the segments are swapped.
- Two-point crossover: Two split points are selected, and the middle chromosome segment is exchanged.

**Figure 6:**
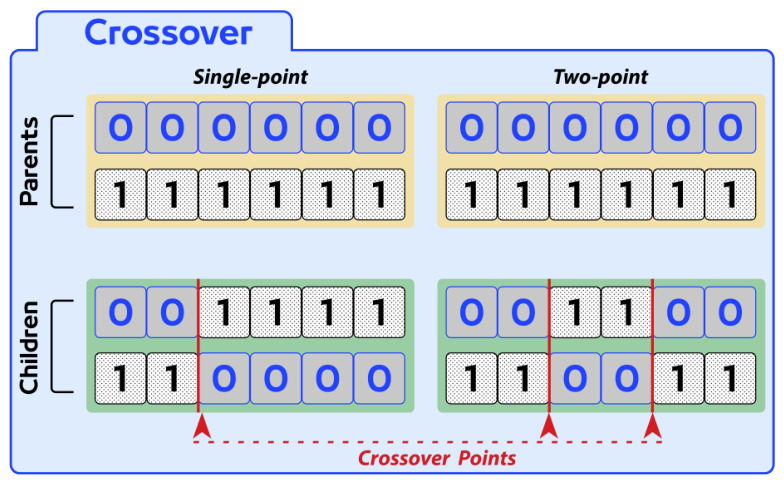
Single-point crossover and two-point crossover in the GA.

These are illustrated in Figure 6. Other crossover variants include multipoint, partially matched, uniform, and half-uniform [31].

#### 2.4.4. Mutation

**Figure 7:**
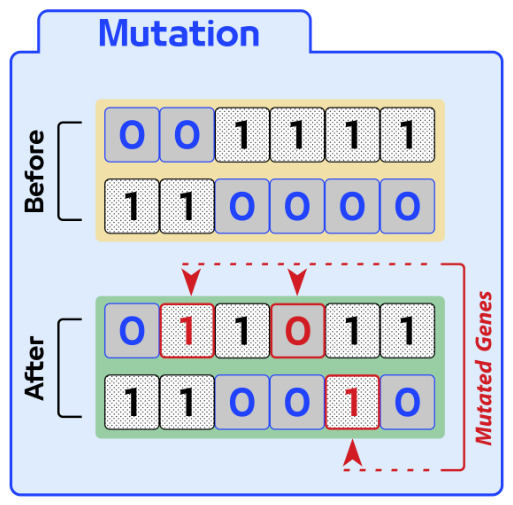
Effect of mutation in the GA.

Crossover only exchanges genetic material and does not directly modify gene values, which may result in a loss of diversity and increased risk of premature convergence. To address this problem, the mutation operator randomly alters genes according to the mutation rate ($M_{r}$). In practice, for each gene, if a random number r∈[0,1] is less than $M_{r}$, the gene is mutated (e.g., by flipping its bit value in binary GA or assigning a new random value in a real-coded GA) [32]. Figure 7 shows this effect. Other mutation variants include uniform, exchange, and boundary [33].

### 2.5. SA

SA was introduced in 1983 by Kirkpatrick et al. as a metaheuristic to solve complex optimization problems [34]. The technique is inspired by metallurgy, where a material is heated and then cooled to achieve a minimum energy state. In optimization, this translates to beginning with a high “temperature” to enable substantial random changes (exploration) and then cooling to reach a stable solution (exploitation). SA uses a probabilistic acceptance rule known as the Metropolis criterion [35], which allows the algorithm to occasionally accept a worse solution, which may help it escape local minima and keep exploring promising regions of the search space [36].

The cooling schedule and acceptance probability [P(accept)] are the main features of SA. The acceptance probability is determined by the temperature *T*, and the cooling rate α (0<α<1) determines how *T* is reduced over iterations. As such, the acceptance probability of a new solution, even if it is worse than the current solution, is given by Equation  (1) [36].

(1) $$P\left(\mathrm{accept}\right)=\left\{\begin{array}{cc} 1 & \mathrm{if}\;\Delta f\leq 0\\ \exp\left(\frac{-\Delta f}{T}\right) & \mathrm{if}\;\Delta f>0 \end{array}\right.$$

**Figure 8:**
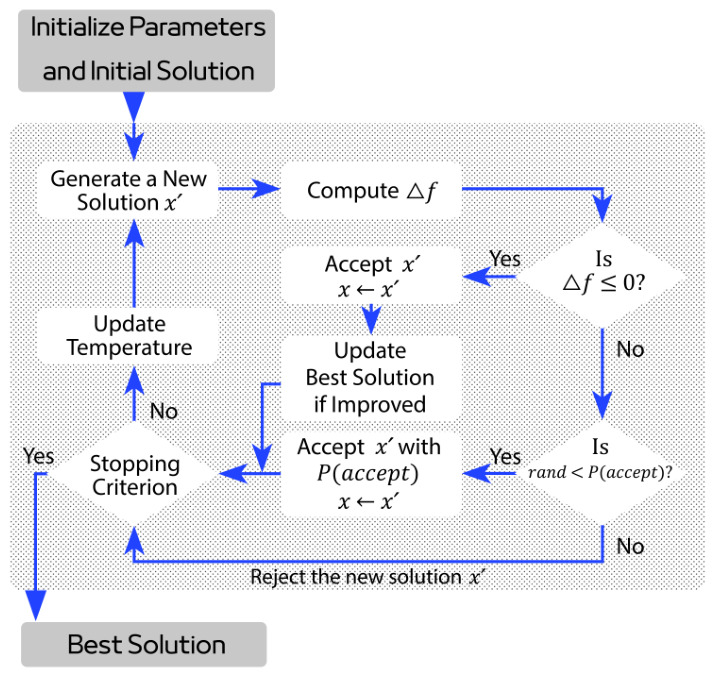
Flowchart of typical SA (minimization).

The difference in cost between the new candidate solution $x^{\prime }$ and the current solution *x* is $\Delta f=f\left(x^{\prime }\right)-f\left(x\right)$. The new candidate solution is obtained by applying a perturbation Δx to the current solution, such that $x^{\prime }=x+\Delta x$. As *T* is lowered, the search becomes more exploitative. The flow diagram of SA is given in Figure 8, and the pseudocode is given in Algorithm 2.
**Algorithm 2** Typical SA (minimization).     **Input:** Initial solution *x*, initial temperature $T_{\mathrm{init}}$, cooling rate α, maximum iterations $I_{max}$     **Output:** Best solution found $x_{best}$  1:Initialize best solution: $x_{best}\leftarrow x$  2:Initialize iteration counter: i←0  3:Initialize temperature: $T\leftarrow T_{\mathrm{init}}$  4:**while** $i<I_{max}$ **do**  5:      Generate a neighbor solution $x^{\prime }$  6:      Evaluate cost difference: $\Delta f\leftarrow f\left(x^{\prime }\right)-f\left(x\right)$  7:      Initialize acceptance flag: accept←false  8:      **if** Δf≤0 **then**  9:           accept←true10:      **else**11:           Generate random number: r∼Uniform(0,1)12:           Compute acceptance probability: $P\leftarrow \exp\left(-\frac{\Delta f}{T}\right)$13:           **if** r<P **then**14:                accept←true15:           **end if**16:      **end if**17:      **if** *accept* **then**18:           Accept new solution: $x\leftarrow x^{\prime }$19:           **if** $f\left(x\right)<f\left(x_{best}\right)$ **then**20:                Update best solution: $x_{best}\leftarrow x$21:           **end if**22:      **end if**23:      Decrease temperature: T←α×T24:      Increment iteration counter: i←i+125:**end while**26:**return** $x_{best}$

## 3. Related Work

This section surveys related work from two complementary perspectives. First, it reviews algorithm-based hybrid FS methods, focusing on GA- and SA-based hybrids that are methodologically related to the proposed framework. Second, it reviews dataset-based studies that have been evaluated on IoTID20, Washington University in St. Louis Enhanced Healthcare Monitoring System (WUSTL-EHMS), and Edge-IIoTset (ML).

### 3.1. Algorithm-Based Related Work

Recent studies have increasingly explored hybrid metaheuristics to address the high dimensionality and redundancy of network features in IDS-oriented FS tasks. Therefore, this subsection highlights representative GA- and SA-driven hybrid FS designs.

Hybrid GA-based FS has been increasingly used to explore large combinatorial feature spaces and enhance IDS performance. Vijayanand and Devaraj [37] combined GA operators with a complementary optimizer for wrapper-based IDS FS. Halim et al. [38] used improved GA-based approaches with customized fitness functions and multiple datasets. Kunhare et al. [39] applied GA to obtain reduced feature sets for traditional ML and hybrid classifier setups, such as logistic regression with a decision tree (DT), for IDS. More recently, Hosseini et al. [40] combined GA with the slime mold algorithm in a hybrid FS approach for IDS.

Likewise, recent hybrid SA-based approaches for IDS FS include Prakash et al. [41], who combined SA with improved salp swarm optimization for FS in IoT-based network IDS. Liu et al. [42] combined SA with adaptive differential evolution for IoT-IDS FS. Huang et al. [43] enhanced binary pigeon-inspired optimization with SA and a population decay factor for feature-subset search in network IDS. Lastly, Han et al. [44] integrated SA into binary PSO to enhance feature-subset search for reflective denial-of-service detection.

These studies show that GA- and SA-based hybrid FS approaches can improve the search process by combining broader exploration with local refinement, which may enhance detection performance and reduce feature redundancy. More importantly, the aforementioned studies did not directly investigate a unified GA–SA hybrid FS approach for recent IoT-oriented intrusion-detection scenarios. Moreover, the studies are not directly comparable because of the lack of consistent reporting of selected feature subsets, validation settings, and computational cost; some studies explicitly report runtime or complexity analysis, whereas others focus mainly on detection performance, and some hybrid enhancements may introduce additional computational overhead.

### 3.2. Dataset-Based Related Work

This subsection provides an overview of recent IDS studies that evaluated FS on the three datasets of interest. The aim is to offer a brief, dataset-based summary of the reported FS approaches, subset sizes, and binary-classification accuracy. Table 1 lists the studies considered for each dataset.

For the IoTID20 dataset, the related studies reported different FS strategies with varying classification accuracies. Alkahtani and Aldhyani [45] used PSO to select 20 features and reported 98.20% accuracy with a long short-term memory (LSTM) model. Bhavsar et al. [46] applied Pearson’s correlation coefficient (PCC)-based FS to select 15 features and achieved 99.00% accuracy with a PCC-based convolutional neural network (PCC-CNN) model. Boubertakh et al. [47] used a binary butterfly optimization algorithm (BBOA) wrapper to select 40 features and reported 98.87% accuracy using LSTM. Lastly, Zayed et al. [48] used firefly algorithm (FA)-based FS in a hybrid deep-learning IDS model combining an FA, a convolutional neural network (CNN), and a recurrent neural network (RNN) to select 47 features and achieved 98.20% accuracy.

For WUSTL-EHMS, previous research has reported various FS techniques with varying performance for the selected feature subsets and classifiers. Chaganti et al. [49] used PSO-based FS and obtained eight optimal features, with 96.00% accuracy using a deep neural network (DNN) classifier. Alalhareth and Hong [50] reported results using logistic redundancy coefficient gradual upweighting and mutual information feature selection (LRGU-MIFS), where the highest accuracy was 96.70% with 25 features and fuzzy-based self-tuning LSTM (FST-LSTM). Lazrek et al. [51] used the recursive feature elimination with DT (RFE-DT), where features were removed until eight features were left; this configuration achieved 97.85% accuracy. Lastly, Pinar et al. [52] introduced a random subset FS with correlation feature selection (RSFS + CFS) approach, where 18 features were selected and 96.37% accuracy was achieved using a DNN classifier.

Research on Edge-IIoTset (ML) has also shown variation in FS design and performance. Thiyam and Dey [53] used the Synthetic Minority Oversampling Technique (SMOTE) and PCC filtering with a variance threshold to select 15 features and reported 98.43% accuracy with DT. Rajput et al. [54] used Fisher-score ranking to select 45 features and achieved 94.54% accuracy with a hyperparameter-tuned random forest (RF) model. Thiyam and Dey [55] applied receiver operating characteristic (ROC)-value feature importance with RF to select 15 features, reporting 99.16% accuracy in the study’s comparison with other works. Lastly, Keshavamurthy et al. [56] applied an ensemble FS approach with seven algorithms and demonstrated that XGBoost achieved 97.64% accuracy with 34 features.

The studies reviewed for these three datasets show substantial differences in how FS is defined and reported, from explicit subset selection (wrapper-style) to ranking-, filter-, and preprocessing-based FS. They also vary in the consistency of reporting subset sizes, runtime/resource indicators, and evaluation settings. While some studies report runtime or memory, such reporting is not consistent across the datasets and evaluation settings. Therefore, to facilitate better dataset-level benchmarking and reproducibility, the current experiments adopted a standardized evaluation protocol; we consistently report subset sizes, selected feature lists, experimental settings, and operational-cost measures.

## 4. Methodology

**Figure 9:**
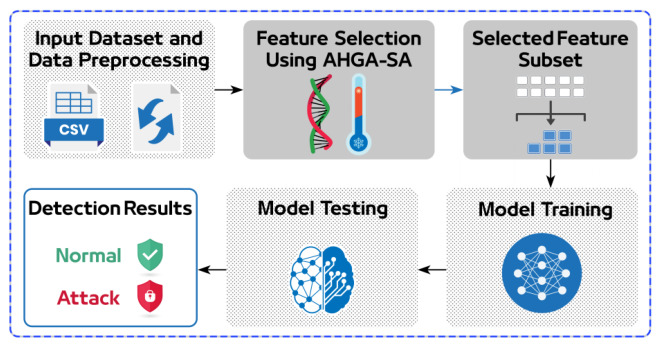
Comprehensive methodology workflow.

**Figure 10:**
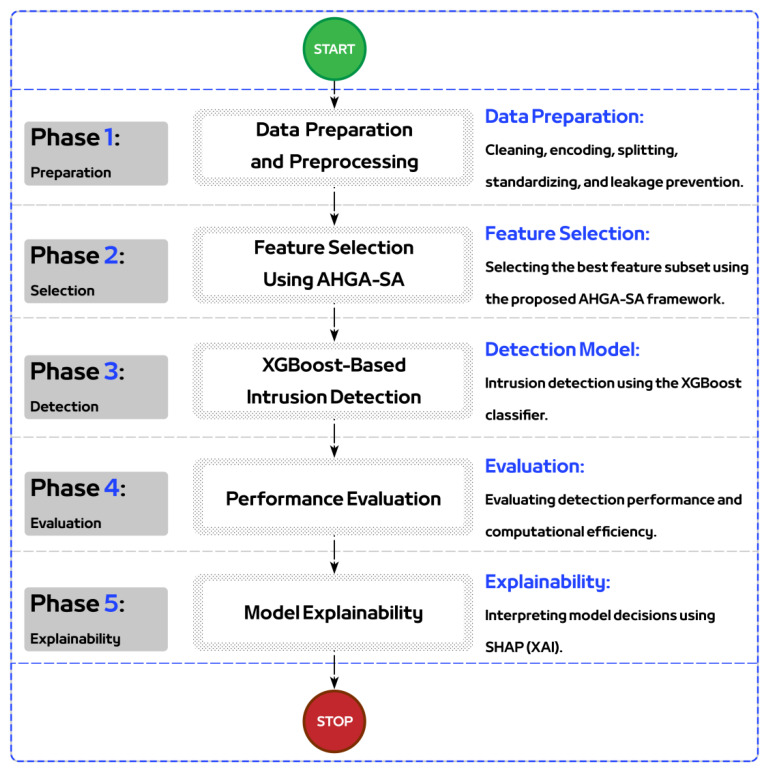
Phase-based research framework.

This section outlines the methodology adopted to design the proposed FS framework for intrusion detection across IoT-oriented datasets. The methodology workflow and the research framework are shown in Figure 9 and Figure 10, respectively.

### 4.1. Data Preprocessing and Benchmark Datasets

The datasets used in this study were preprocessed to enhance data consistency and facilitate the FS and intrusion-detection processes. The preprocessing steps were applied across the datasets and included:

- Infinite-value handling: Positive and negative infinite values were replaced with missing values.
- Data splitting: The data were first split into training and test sets using an 80%/20% split with shuffling and stratification. The training set was then further split into internal training and validation sets via an 80%/20% split with shuffling and stratification for the AHGA-SA search process.
- Feature removal: Potentially leakage-prone features were first examined through internal training/validation diagnostic behavior, with the diagnostic results later discussed in Section 7. The held-out test set was not used to decide feature removal. Before reporting the final test results, the final removal decision was also supported by feature semantics to exclude dataset-specific fields that could encode non-generalizable artifacts, including identifiers, addressing fields, temporal or ordering fields, payload- or content-bearing fields, and protocol-specific or derived flow/session fields. The complete removed-feature lists are reported in the corresponding dataset descriptions.
- Missing-value imputation: Missing numerical values were replaced with the median; the imputer was fitted on the training portion of each split and then applied to the corresponding validation or test portion.
- Categorical encoding: Categorical features were encoded numerically using a label encoder fitted on the training portion of each split and then applied to the corresponding validation or test portion.
- Standardization: Numerical features were standardized using StandardScaler, which was fitted on the training portion of each split and then applied to the corresponding validation or test portion.

#### 4.1.1. IoTID20 Dataset

The IoTID20 dataset [57] was introduced in 2020 by Imtiaz Ullah and Mahmoud [58] to offer a comprehensive IoT botnet benchmark with network- and flow-based features for developing and evaluating flow-based intrusion detection in IoT networks. This dataset contains labeled normal and attack traffic and can be used to detect anomalies in IoT networks.

- Class distribution: 625,783 samples.
  – Label 0 (Normal): 40,073
  – Label 1 (Attack): 585,710
- Feature summary: 83 features + 3 label columns.
  – Total features (Initial): 83
  – Features after removal (Final): 65
- Removed features: 18 features.
  – “Timestamp,” “Active_Max,” “Bwd_IAT_Max,”
    “Src_Port,” “Dst_Port,” “Src_IP,” “Dst_IP,”
    “Subflow_Bwd_Byts,” “Subflow_Bwd_Pkts,”
    “Subflow_Fwd_Byts,” “Subflow_Fwd_Pkts,”
    “Bwd_Seg_Size_Avg,” “Fwd_IAT_Max,”
    “PSH_Flag_Cnt,” “Pkt_Size_Avg,”
    “Fwd_Seg_Size_Avg,” “Idle_Max,”
    “Flow_ID”
- Data splitting: 80% for training and 20% for testing.
  – Training set (500,626 samples):
    32,168 for Normal, 468,458 for Attack.
  – Test set (125,157 samples):
    7905 for Normal, 117,252 for Attack.

#### 4.1.2. WUSTL-EHMS Dataset

In 2020, Hady et al. [59] created the WUSTL-EHMS dataset [60] to enhance intrusion detection in IoT-based healthcare systems. Generated using man-in-the-middle attacks, it includes network-flow features and patient biometric data. The dataset was cleaned and labeled with attack types and binary labels for ML applications.

- Class distribution: 16,318 samples.
  – Label 0 (Normal): 14,272
  – Label 1 (Attack): 2046
- Feature summary: 43 features + 2 label columns.
  – Total features (Initial): 43
  – Features after removal (Final): 36
- Removed features: 7 features.
  – “Dir,” “Flgs,” “SrcAddr,” “DstAddr,” “SrcMac,”
    “DstMac,” “Packet_num”
- Data splitting: 80% for training and 20% for testing.
  – Training set (13,054 samples):
    11,372 for Normal, 1682 for Attack.
  – Test set (3264 samples):
    2900 for Normal, 364 for Attack.

#### 4.1.3. Edge-IIoTset (ML) Dataset

The Edge-IIoTset (ML) dataset [61], proposed by Ferrag et al. [62] in 2022, is a realistic cybersecurity benchmark for IoT and IIoT applications that can be used in both centralized and federated learning scenarios. It was created using a multilayer IoT/IIoT testbed with a variety of device types and contains labeled traffic with 14 attacks in five major threat categories, allowing for the development and evaluation of ML-based IDSs. The ML version was used in this study.

- Class distribution: 157,800 samples.
  – Label 0 (Normal): 24,301
  – Label 1 (Attack): 133,499
- Feature summary: 61 features + 2 label columns.
  – Total features (Initial): 61
  – Features after removal (Final): 42
- Removed features: 19 features.
  – “dns.qry.name.len,” “tcp.payload,” “tcp.srcport,”
    “mqtt.protoname,” “icmp.transmit_timestamp,”
    “http.request.full_uri,” “arp.dst.proto_ipv4,”
    “http.file_data,” “mqtt.topic,” “mqtt.msg,”
    “frame.time,” “ip.src_host,” “tcp.options,”
    “mqtt.conack.flags,” “arp.src.proto_ipv4,”
    “http.request.uri.query,” “ip.dst_host,”
    “udp.port,” “tcp.dstport”
- Data splitting: 80% for training and 20% for testing.
  – Training set (126,240 samples):
    19,471 for Normal, 106,769 for Attack.
  – Test set (31,560 samples):
    4830 for Normal, 26,730 for Attack.

Table 2 summarizes the initial, removed, and final feature counts for each dataset.

### 4.2. AHGA-SA

The proposed approach uses AHGA-SA for FS, which integrates the global search ability of GA and the local exploitation ability of SA. To prevent selection leakage, the data are first split into training and test sets, represented as ($X_{\mathrm{train}}$, $y_{\mathrm{train}}$) and ($X_{\mathrm{test}}$, $y_{\mathrm{test}}$), respectively. In the AHGA-SA search process, the training set is further split into an internal training subset ($X_{\mathrm{int}\_\mathrm{train}}$, $y_{\mathrm{int}\_\mathrm{train}}$) and a validation subset ($X_{\mathrm{val}}$, $y_{\mathrm{val}}$). The internal training subset is used to train candidate feature subsets, and the validation subset is used to evaluate them, while the test set is only used for final testing.

A GA begins with a binary population, with “1” and “0” representing selected and unselected features, respectively. A GA searches the solution space through repeated selection, crossover, and mutation but can still become trapped in local optima. To overcome this issue, SA is used after crossover and mutation to generate neighboring solutions. Better solutions are always accepted, while worse solutions are accepted with a certain probability based on the Metropolis criterion, enabling the search to escape local optima. The temperature is gradually reduced to shift the search from exploration to exploitation.

Prior to the hybrid optimization, XGBoost is first trained with only two estimators to obtain a quick initial importance ranking, and only features with importance scores greater than zero are retained to reduce the search space for the subsequent AHGA-SA search. After this preselection step, AHGA-SA is executed in two search stages: the first search stage explores the reduced feature space and keeps track of the feature counts associated with the retained best solutions, and the second search stage restricts the search to these selected feature counts. This makes the second search stage more targeted by focusing on subset sizes that are found to be promising in the first search stage. The best solution is replaced if a new candidate has a higher validation accuracy or the same validation accuracy but fewer features.

Adaptive tournament selection also controls the balance between exploration and exploitation based on the SA temperature: higher temperatures encourage diversity, while lower temperatures encourage fitter individuals and convergence. This adaptive approach allows the complementary roles of GA exploration and SA exploitation to navigate the search space and generate small, informative feature subsets. The following are the main steps and key design considerations of the AHGA-SA framework:

- Initial setup:
  – Input variables: The framework uses an internal training subset and a validation subset derived from the training data. The held-out test set is exclusively reserved for final evaluation.
  – Parameters: These include population size (*N*), fixed two-stage AHGA-SA search design $\left(S_{\max}=2\right)$, crossover rate ($C_{r}$), mutation rate ($M_{r}$), tournament size (*S*), initial temperature (*T*), and cooling rate (α). Together, they regulate the genetic and annealing operations and help strike a balance between exploration and exploitation.
- Core functional steps:
  – XGBoost feature preselection: As a lightweight preselection step to obtain a rapid initial feature ranking, XGBoost is trained on the internal training subset using two estimators. Features with importance scores greater than zero are retained, thereby reducing the search space.
  – Population initialization: The population is initialized by generating random binary vectors, where 1 and 0 denote selected and unselected features, respectively, from the subset preselected by XGBoost.
  – Duplicate-removal strategy: To maximize diversity and reduce redundant computation, duplicates are checked and removed after population initialization and after hybridization.
  – Fitness evaluation: In each search stage, the fitness of each individual is evaluated on the internal training and validation subsets at three evaluation points—after duplicate removal, after offspring generation, and after SA execution—to capture any improvement achieved at each stage.
  – Adaptive tournament selection: To support adaptive exploration and exploitation, tournament selection pressure is adjusted according to the current SA temperature.
  – Hybrid SA: SA generates neighboring solutions through further refinement; new solutions are accepted if they improve fitness or, if they are worse, probabilistically based on the temperature-dependent acceptance criterion.
  – Temperature cooling: Gradual temperature reduction shifts the search from broader exploration toward exploitation and convergence.
- Best-solution update and stage-based refinement:
  – Best-solution update: The first evaluated solution is stored as the initial entry in the best-solution list. Thereafter, the list is updated whenever a new candidate achieves a higher validation accuracy or the same validation accuracy but fewer features. Each retained entry stores the corresponding feature subset and validation accuracy.
  – First search stage: Broadly explores the preselected feature space to identify promising subsets.
  – Second search stage: Refines the search by exploring subsets with the same feature counts as the retained best solutions from the first search stage.
- Termination and output:
  – Termination: The search process stops after completing the predefined AHGA-SA search stages.
  – Output: The best feature subset obtained during the search process is returned as the final solution.

Algorithm 3 presents the pseudocode of the proposed AHGA-SA, summarizing its main steps.

**Algorithm 3** AHGA-SA algorithm for feature selection.
     **Input:** $X_{\mathrm{int}\_\mathrm{train}}$, $y_{\mathrm{int}\_\mathrm{train}}$, $X_{\mathrm{val}}$, $y_{\mathrm{val}}$, population size *N*, tournament size *S*, crossover rate $C_{r}$, mutation rate $M_{r}$, cooling rate α, initial temperature $T_{\mathrm{init}}$     **Output:** Best obtained feature subset $x_{\mathrm{best}}$  1:Compute feature-importance scores using two-estimator XGBoost  2:Select features k←{featureswithimportance>0}  3:Restrict $X_{\mathrm{int}\_\mathrm{train}}$ and $X_{\mathrm{val}}$ to the features in *k*  4:Initialize population *P* with *N* random binary subsets (non-empty)  5:Initialize best fitness $f_{\mathrm{best}}\leftarrow -\infty$  6:Initialize best subset $x_{\mathrm{best}}\leftarrow \left[\;\right]$  7:Initialize list of best solutions $s_{\mathrm{best}}\leftarrow \left[\;\right]$  8:Initialize list of feature counts of best solutions $c_{\mathrm{best}}\leftarrow \left[\;\right]$  9:Set number of search stages $S_{\max}\leftarrow 2$, temperature $T\leftarrow T_{\mathrm{init}}$10:Remove duplicates from *P*; evaluate fitness of all individuals in *P*11:**if** any individual has higher fitness (tie-break: fewer features) **then**12:      Update $f_{\mathrm{best}},x_{\mathrm{best}}$13:      Append $x_{\mathrm{best}}$ to $s_{\mathrm{best}}$ and its feature count to $c_{\mathrm{best}}$14:
**end if**
15:**for** stage=1 **to** $S_{\max}$ **do**16:       **if** stage==2 **then**17:             $C^{⋆}\leftarrow D$ istinct $\left(c_{\mathrm{best}}\right)$18:             Reinitialize *P* with *N* (non-empty) using feature counts from $C^{⋆}$19:             Remove duplicates from *P*; evaluate fitness of all individuals in *P*20:             **if** any individual has higher fitness (tie-break: fewer features) **then**21:                  Update $f_{\mathrm{best}},x_{\mathrm{best}}$22:                  Append $x_{\mathrm{best}}$ to $s_{\mathrm{best}}$ and its feature count to $c_{\mathrm{best}}$23:            **end if**24:      **end if**25:      Initialize offspring←[]26:      **while** |offspring|<N **do**27:            Select {parent1,parent2} via adaptive tournament selection (*S*, *T*)28:            Apply crossover with probability $C_{r}$ to generate {child1,child2}29:            Apply mutation to {child1,child2} with probability $M_{r}$30:            **if** stage==2 **then**31:                  Enforce each offspring’s feature count to be in $C^{⋆}$32:            **end if**33:            Append {child1} to offspring34:            **if** |offspring|<N **then**35:                 Append {child2} to offspring36:            **end if**37:      **end while**38:      **for all** child∈offspring **do**39:            Evaluate fitness of child $f_{\mathrm{child}}$40:            Generate a neighbor via a local bit-swap mutation41:            Evaluate fitness of neighbor $f_{\mathrm{neighbor}}$42:            **if** $f_{\mathrm{neighbor}}\geq f_{\mathrm{child}}$ **then**43:                 Accept the neighbor; set child←neighbor and $f_{\mathrm{child}}\leftarrow f_{\mathrm{neighbor}}$44:            **else**45:                 $P_{\mathrm{accept}}\leftarrow \min\;\left(1,\;\exp\;\left(\frac{f_{\mathrm{neighbor}}-f_{\mathrm{child}}}{T}\right)\right)$46:                 **if** $P_{\mathrm{accept}}>\mathrm{Uniform}\left(0,1\right)$ **then**47:                      Accept the neighbor; set child←neighbor and $f_{\mathrm{child}}\leftarrow f_{\mathrm{neighbor}}$48:                 **end if**49:            **end if**50:      **end for**51:      **if** any offspring has higher fitness (tie-break: fewer features) **then**52:            Update $f_{\mathrm{best}},x_{\mathrm{best}}$53:            Append $x_{\mathrm{best}}$ to $s_{\mathrm{best}}$ and its feature count to $c_{\mathrm{best}}$54:      **end if**55:      **if** stage==1 **then**56:           Remove duplicates from offspring57:           Replace *P* with offspring58:           Decrease temperature: T←α×T59:      **end if**60:
**end for**
61:**Return** $x_{\mathrm{best}}$


### 4.3. Detection Model

We employed an XGBoost classifier in the detection models. XGBoost is a scalable gradient boosting algorithm introduced by Chen and Guestrin in 2016, with competitive predictive performance, computational efficiency, and a regularized learning objective to reduce overfitting [63]. Based on these characteristics, it was selected for the classification tasks.

### 4.4. Model Evaluation

The classifiers were evaluated based on their ability to distinguish attack traffic from normal traffic using a comprehensive set of metrics. Additionally, operational metrics were considered to assess computational efficiency and resource usage [64,65,66].

**Figure 11:**
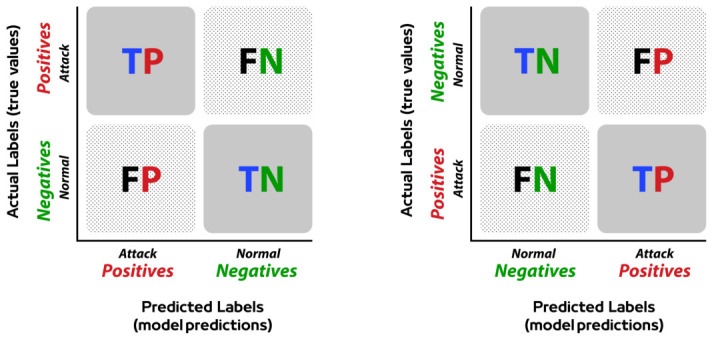
CM layouts in binary intrusion detection.

- Confusion matrix (CM): The CM is a table that compares the actual and predicted classes and summarizes classification outcomes. As shown in Figure 11, the matrix layout depends on the definition of the positive class; “Attack” was defined as the positive class. The CM consists of four components:
  – True positives (TP): Attack instances correctly classified as attacks.
  – True negatives (TN): Normal instances correctly classified as normal.
  – False positives (FP): Normal instances incorrectly classified as attacks.
  – False negatives (FN): Attack instances incorrectly classified as normal.
- Performance metrics:
  – Accuracy (Acc): The proportion of correctly classified samples among all samples.
  – Matthews correlation coefficient (MCC): A correlation-based measure computed from TP, TN, FP, and FN. It ranges from −1 to +1 and is particularly suitable for imbalanced datasets.
  – Precision or positive predictive value (PPV): The proportion of predicted attacks that are truly attacks.
  – Recall or sensitivity or true positive rate (TPR): The proportion of actual attacks that are correctly detected.
  – Specificity or true negative rate (TNR): The proportion of normal traffic that is correctly identified.
  – F1 score: The harmonic mean of precision and recall.
  – FPR or Type I error: The proportion of normal traffic incorrectly classified as attacks.
  – FNR or Type II error: The proportion of attacks incorrectly classified as normal. This metric is particularly important, as it indicates missed attacks.
  These metrics, computed using the formulas in Table 3, provide an overall assessment of model performance [67].
- Threshold-independent evaluation:
  – Area under the ROC curve (AUC-ROC): This metric measures the model’s ability to distinguish between the positive class, “Attack,” and the negative class, “Normal,” across all possible threshold values. Unlike CM-based metrics at a fixed threshold, AUC-ROC was computed from prediction probabilities across different classification thresholds. Higher AUC-ROC values indicate better overall discriminative performance.
  – ROC curve: It is constructed from prediction probabilities by plotting the TPR against the FPR across different classification thresholds. It visually represents the trade-off between detection sensitivity and false alarm rate.
- Operational metrics:
  – FS process time: The total end-to-end time required to complete the FS process for the baseline FS method and AHGA-SA.
  – Number of features: The number of features selected and used for model training and testing.
  – Training time: The time required to fit the model on the training set.
  – Testing time: The time required for the model to generate predictions on the test set.
  – Central processing unit (CPU) usage: CPU utilization was separately recorded during model training and testing via Windows Performance Recorder, with the traces analyzed using Windows Performance Analyzer.
  – Resident set size (RSS) overhead: The increase in process memory consumption, measured as the difference between the peak process RSS recorded using the psutil library during each phase and the baseline RSS immediately before that phase.
  For operational measurements, one warm-up run was performed and excluded from reporting, followed by five consecutive measured runs within the same measurement session under the same data split, selected feature subset, random states, and classifier configuration. Non-essential background applications were closed during measurement. The training/testing time, CPU-usage, and RSS-overhead values reported in the main result tables represent the mean of the five measured runs, while the corresponding standard deviations are provided in Appendix A.

### 4.5. Experimental Setup

To ensure reproducibility, all experiments were performed in a controlled computational environment. The hardware and software specifications are listed in Table 4, while Table 5 presents the algorithm parameters and programming settings.

Considering the dataset diversity and available computational resources, these configurations were selected to support effective FS and classification in a consistent experimental setting; the algorithm parameter choices are further discussed in Section 7. RF was used as the baseline FS method, as it provides feature-importance estimates, is robust to noise and overfitting, and can perform well in high-dimensional settings, thereby making it a practical baseline for comparison [68,69]. XGBoost was employed for feature preselection and for model training and testing. As shown in Table 5, the number of estimators was set on a dataset-specific basis, as the datasets differed in their characteristics and computational burden. Moreover, SHAP was used to support the interpretation of the resulting XGBoost model.

## 5. Results

The next subsections show the RF-based FS baseline results and corresponding model results, followed by the AHGA-SA FS results and final model results across the three datasets.

### 5.1. IoTID20 Experimental Results

The IoTID20 results are presented first.

#### 5.1.1. Baseline RF-Based FS—IoTID20

**Figure 12:**
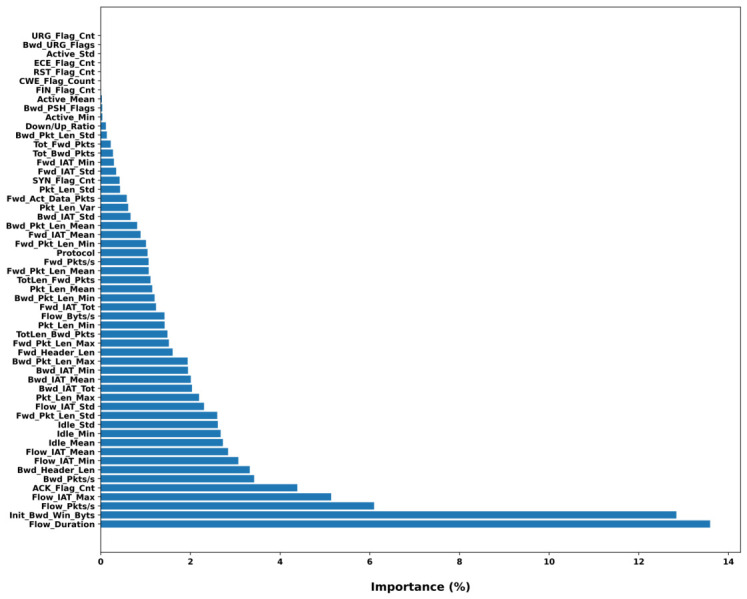
Top RF feature-importance scores—IoTID20.

For the IoTID20 dataset, the RF classifier was used to calculate feature-importance scores for the 65 features. A total of 55 features were retained after removing features with zero importance. The scores are shown in Table 6, and the top features are shown in Figure 12. The FS processing time was 34,702 milliseconds (ms).

#### 5.1.2. Baseline Model Performance—IoTID20

**Figure 13:**
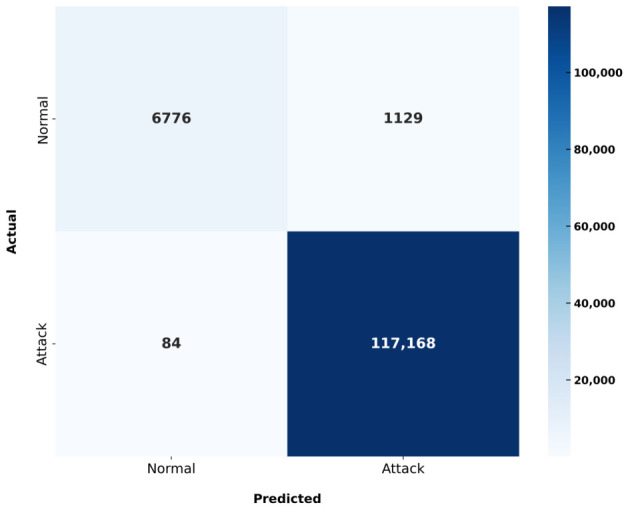
CM for the baseline model—IoTID20.

The baseline model was evaluated with the XGBoost classifier using the 55 features selected in the previous step. The performance metrics are presented in Table 7, and the CM is illustrated in Figure 13.

#### 5.1.3. Proposed FS Method (AHGA-SA)—IoTID20

The XGBoost preselection process was a lightweight filtering stage that retained 31 of the 65 features with importance scores greater than zero in 137 ms, as listed in Table 8.

**Figure 14:**
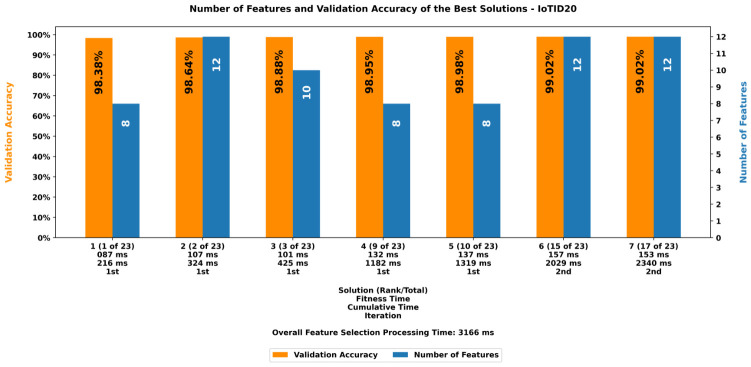
Best AHGA-SA solutions for the IoTID20 dataset.

**Figure 15:**
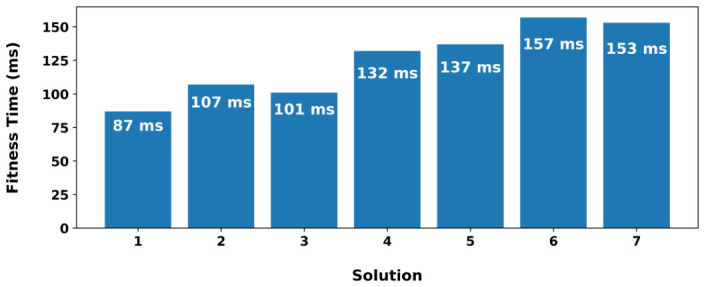
Fitness time for the best solutions—IoTID20.

**Figure 16:**
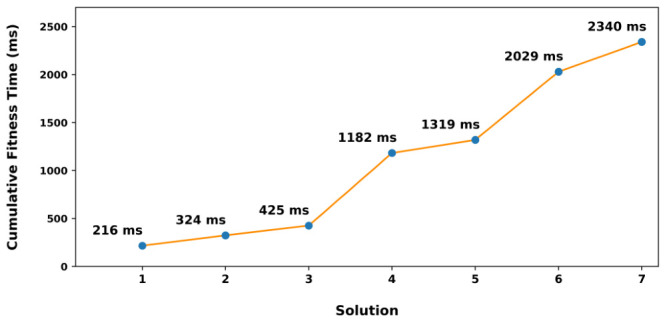
Cumulative time for the best solutions—IoTID20.

AHGA-SA then searched for effective feature combinations from that subset. With a population size of four, two search stages, and three evaluation points per search stage, the search produced 24 solutions, resulting in 23 unique solutions after one duplicate was removed by the framework’s duplicate-removal strategy. The retained best solutions are shown in Figure 14, with the solution ranks calculated among these 23 unique solutions. The orange bars show the validation accuracy of the displayed solutions, and the blue bars show the number of features. The solutions are shown in the order in which they were retained. Since the list of best solutions is initially empty, the first solution is retained as the initial best solution. Subsequently, a solution is retained only if it is better than the previously retained solution according to the framework criterion, which favors solutions with higher validation accuracy and, in the case of ties, with fewer features. For each solution shown, the figure also lists its solution number and rank out of the 23 unique solutions, its fitness time, cumulative time from the beginning of the AHGA-SA search, and the search stage in which it was found. Therefore, the last solution retained, No. 7, is the final best solution, which is found in the second search stage, where the search focuses on subsets with the same feature counts as the retained best solutions in the first search stage. The subsets of selected features are given in Table 9. Figure 15 and Figure 16 report the fitness time and cumulative time of the best solutions, respectively.

#### 5.1.4. Final Model Performance—IoTID20

**Figure 17:**
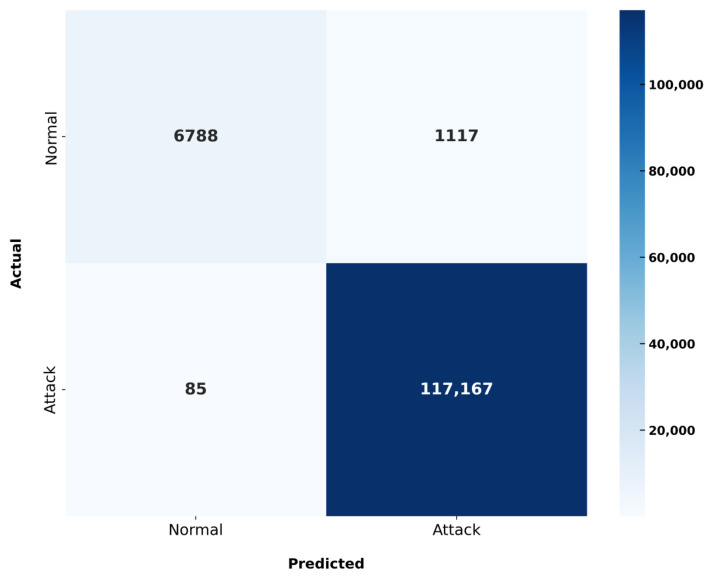
CM for the final model—IoTID20.

**Figure 18:**
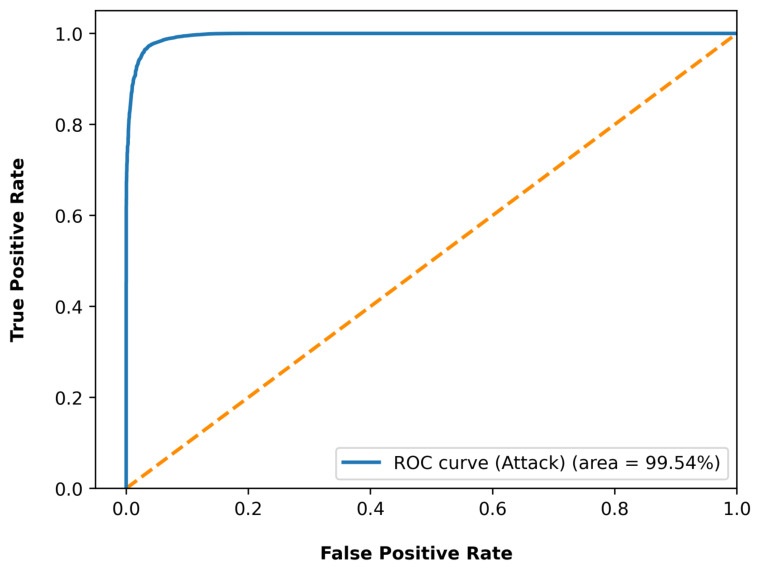
ROC curve of the final model—IoTID20.

The final model was evaluated with the XGBoost classifier on the 12-feature subset selected by AHGA-SA. The results are shown in Table 10. The CM is shown in Figure 17, and the ROC curve is shown in Figure 18.

### 5.2. WUSTL-EHMS Experimental Results

Next, the results for the WUSTL-EHMS dataset are shown.

#### 5.2.1. Baseline RF-Based FS—WUSTL-EHMS

**Figure 19:**
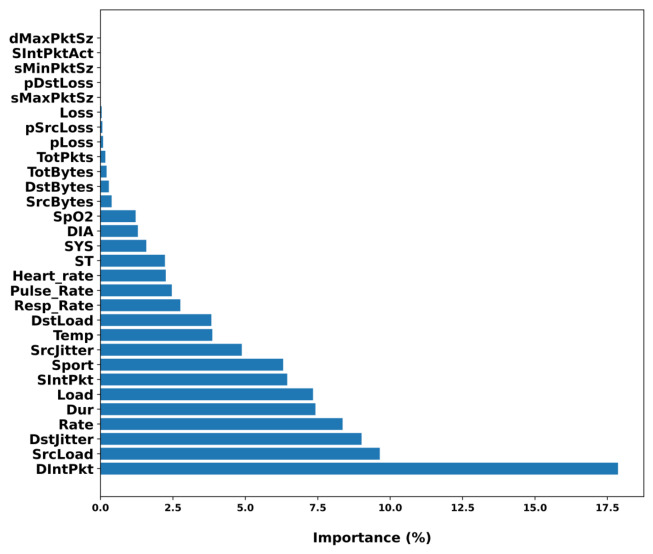
Top RF feature-importance scores—WUSTL-EHMS.

For the WUSTL-EHMS dataset, the RF classifier was used to calculate feature-importance scores for the 36 features. After removing features with zero importance, 30 features were retained in 1254 ms. The scores are shown in Figure 19.

#### 5.2.2. Baseline Model Performance—WUSTL-EHMS

**Figure 20:**
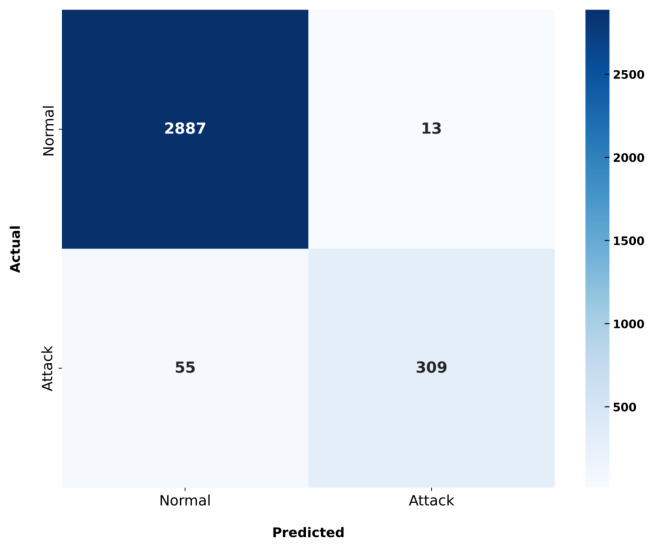
CM for the baseline model—WUSTL-EHMS.

The baseline model was evaluated with the XGBoost classifier using the 30-feature subset. Table 11 shows the performance metrics, and Figure 20 shows the CM.

#### 5.2.3. Proposed FS Method (AHGA-SA)—WUSTL-EHMS

**Figure 21:**
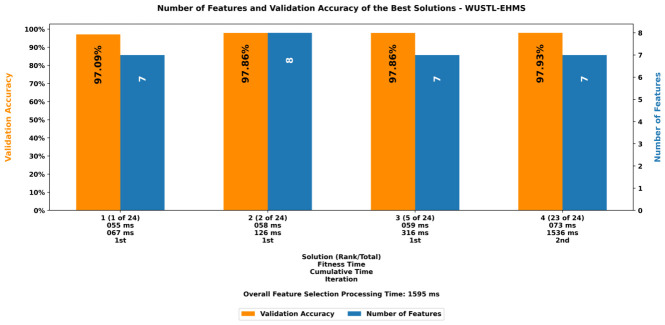
Best AHGA-SA solutions for the WUSTL-EHMS dataset.

The XGBoost preselection step retained 13 features out of the 36 features with importance scores greater than zero in 10 ms, as listed in Table 12. AHGA-SA was then applied to the preselected features, and the best solution selected seven features: “DIntPkt,” “Pulse_Rate,” “Resp_Rate,” “SYS,” “Heart_rate,” “Sport,” and “Temp.” The results for AHGA-SA are shown in Figure 21.

#### 5.2.4. Final Model Performance—WUSTL-EHMS

**Figure 22:**
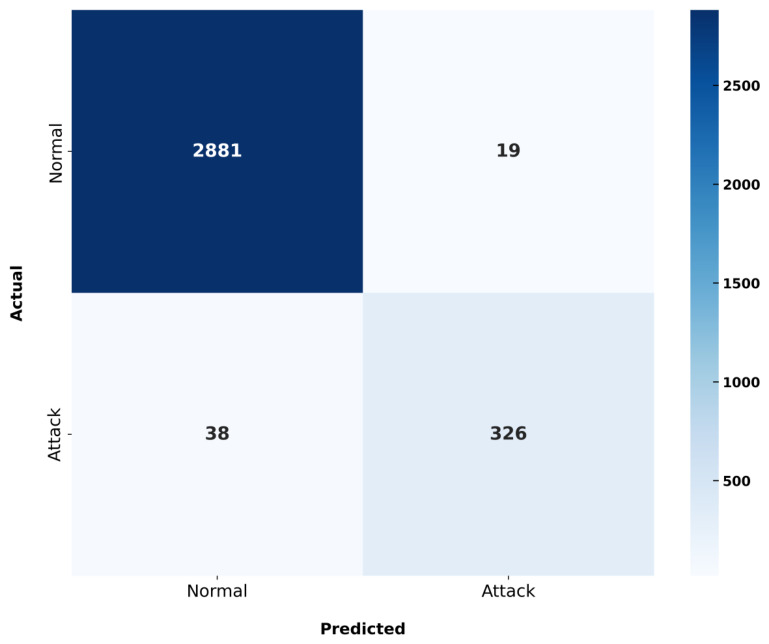
CM for the final model—WUSTL-EHMS.

The final model was evaluated with the XGBoost classifier using the seven features selected by AHGA-SA. The performance metrics are shown in Table 13, and the CM is shown in Figure 22.

### 5.3. Edge-IIoTset (ML) Experimental Results

Finally, we report the results for Edge-IIoTset (ML).

#### 5.3.1. Baseline RF-Based FS—Edge-IIoTset (ML)

**Figure 23:**
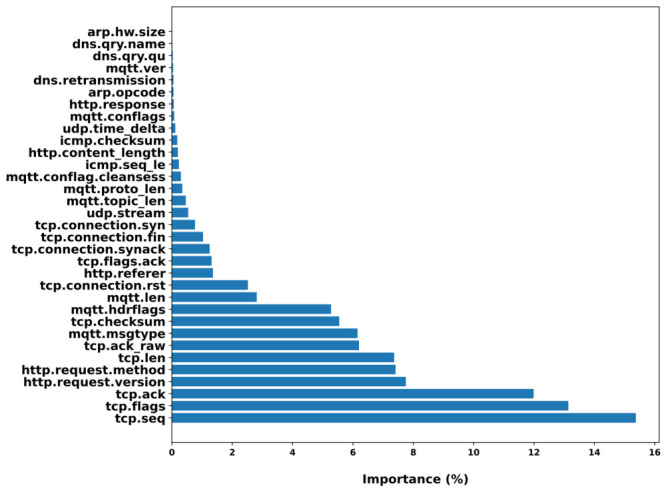
Top RF feature-importance scores—Edge-IIoTset (ML).

For the Edge-IIoTset (ML) dataset, we used the RF classifier to calculate the feature-importance scores for the 42 features. A total of 33 features were retained in 3091 ms after removing features with zero importance. The scores are shown in Figure 23.

#### 5.3.2. Baseline Model Performance—Edge-IIoTset (ML)

**Figure 24:**
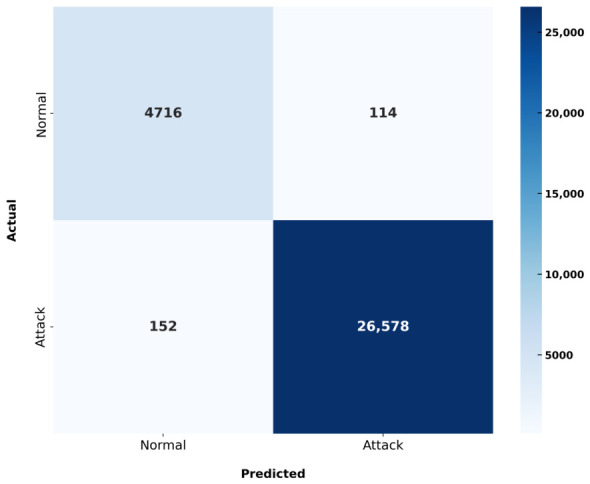
CM for the baseline model—Edge-IIoTset (ML).

The baseline model was evaluated on the 33-feature set using the XGBoost classifier. The performance metrics are shown in Table 14, and the CM is shown in Figure 24.

#### 5.3.3. Proposed FS Method (AHGA-SA)—Edge-IIoTset (ML)

**Figure 25:**
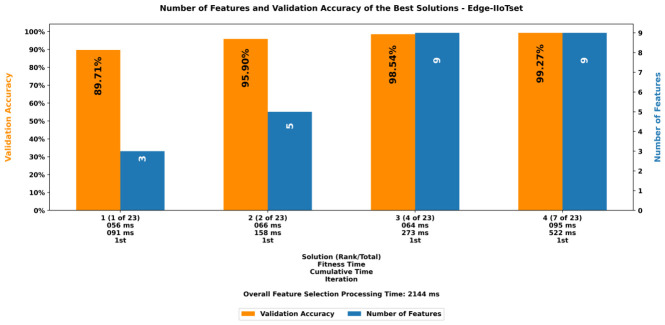
Best AHGA-SA solutions for the Edge-IIoTset (ML) dataset.

The XGBoost preselection step reduced the feature space from 42 to 11 in 29 ms, as listed in Table 15. AHGA-SA was then used to select the best features from that subset, and the best solution contained seven features: “tcp.checksum,” “http.request.version,” “udp.time_delta,” “tcp.seq,” “tcp.flags,” “tcp.ack,” and “tcp.ack_raw.” The AHGA-SA results are presented in Figure 25.

#### 5.3.4. Final Model Performance—Edge-IIoTset (ML)

**Figure 26:**
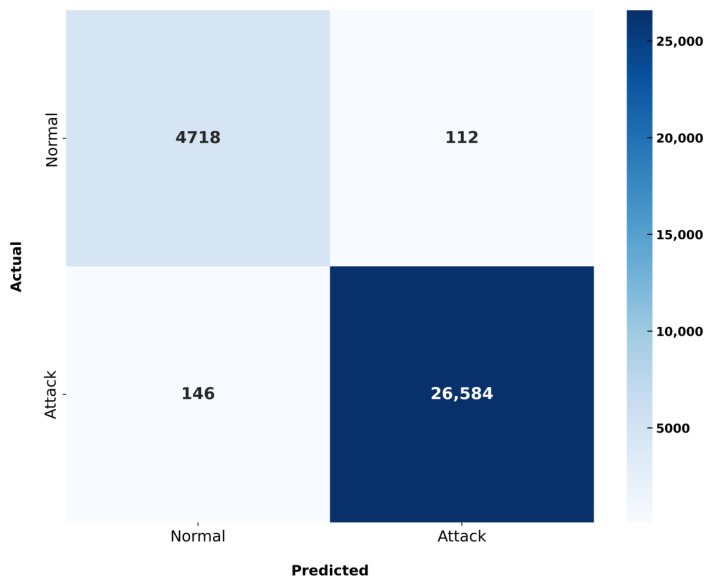
CM for the final model—Edge-IIoTset (ML).

The final model was evaluated using the XGBoost classifier with the seven features selected by AHGA-SA. The performance metrics are shown in Table 16, while the CM is shown in Figure 26.

## 6. XAI

SHAP is derived from the Shapley value, a game theory concept proposed by Lloyd Shapley in 1953 [70] and later adapted into a practical unified explanation framework by Lundberg and Lee [71]. It provides a contribution value for each feature, which can be used to explain how features contribute to model predictions and thus enhance the interpretability of black-box models [72,73].

SHAP was applied to explain how the final XGBoost model made predictions using the seven-feature subset identified by AHGA-SA; the WUSTL-EHMS dataset was chosen for this illustrative example. This dataset was selected because the small subset size enabled easier visualization, and the results showed the greatest improvement over the baseline among the three datasets. For clarity in the binary setting, the SHAP visualizations are shown for the positive class, “Attack.”

### 6.1. SHAP Beeswarm Plot

**Figure 27:**
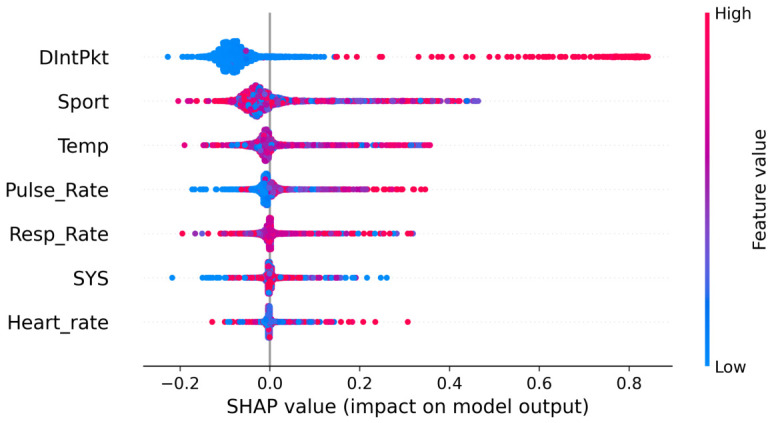
SHAP beeswarm plot—WUSTL-EHMS.

The SHAP beeswarm plot offers a compact global view of how the most important features affect the model output for the test set. In Figure 27, features are ranked by the mean absolute SHAP value; each dot corresponds to a test instance, and the dot’s color indicates the original feature value (blue is low, red is high). Positive SHAP values increase the prediction for the class shown, while negative values decrease it. We used Tree SHAP with XGBoost to explain all 3264 test instances of the WUSTL-EHMS dataset. The beeswarm plot for the “Attack” class is shown in Figure 27. The most important feature is “DIntPkt,” with higher values tending to push the prediction toward attack and lower values shifting it away.

### 6.2. SHAP Violin Plot

**Figure 28:**
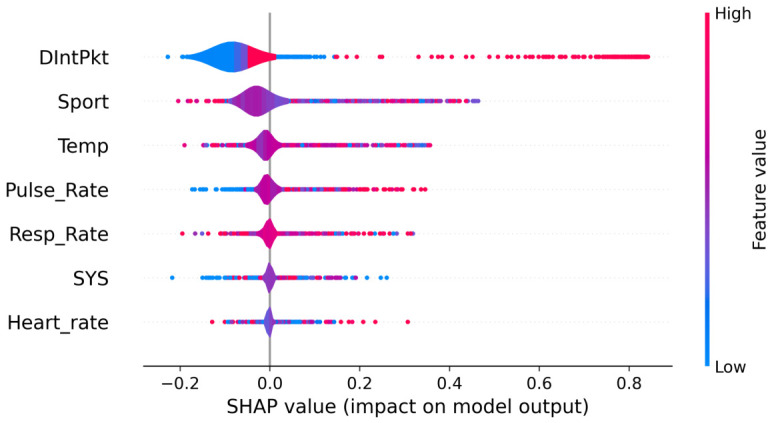
SHAP violin plot—WUSTL-EHMS.

The violin plot in Figure 28 complements the beeswarm plot, as it displays the density of the SHAP values for each feature across the test set. This can help identify the spread and concentration of feature contributions to the prediction of the “Attack” class.

### 6.3. SHAP Waterfall Plot

**Figure 29:**
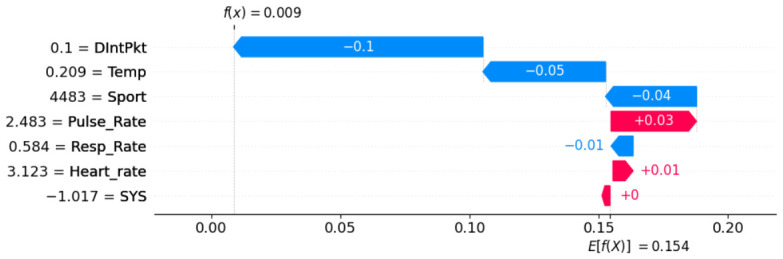
SHAP waterfall plot—WUSTL-EHMS.

The SHAP waterfall plot shows how the selected features contribute to the prediction of a single instance. It begins with the expected value E[f(x)], which is the model’s output, before taking into account the feature values of the selected instance, and then it displays the way in which positive and negative SHAP contributions move the prediction toward the final model output f(x). The number before each feature name is the actual value of the feature in the selected instance. As shown in Figure 29, the largest SHAP contributions were from the features “DIntPkt,” “Temp,” and “Sport,” which all contributed to moving the model output away from the “Attack” class, while “Pulse_Rate” contributed a smaller positive value. These contributions led to a model output of f(x)=0.009, which is a very low probability of attack.

### 6.4. SHAP Force Plot

**Figure 30:**
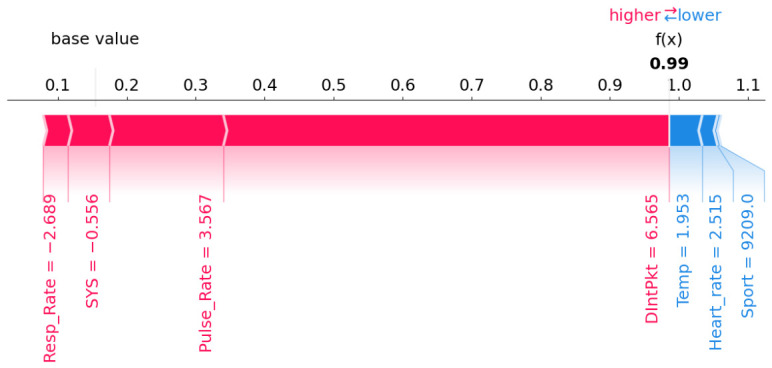
SHAP force plot—WUSTL-EHMS.

The SHAP force plot also provides an explanation for the prediction of one instance by visualizing the influence of the selected features on the model output. It begins with the baseline, which is the model output without taking into account the feature values of the instance. The features then push the model output up or down for the class shown (red features push up, blue features push down). These effects add up to the model output f(x). In Figure 30, the features “DIntPkt,” “Pulse_Rate,” “SYS,” and “Resp_Rate” pushed the prediction toward the “Attack” class, resulting in f(x)=0.99, which indicated weak support for the “Normal” class.

### 6.5. SHAP Decision Plot

**Figure 31:**
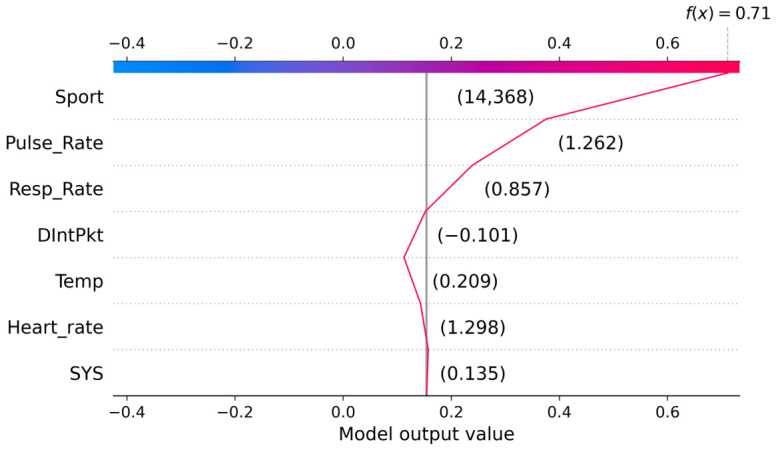
SHAP decision plot for single instance—WUSTL-EHMS.

The SHAP decision plot illustrates how the model output varies from the expected value as the feature contributions are added from the bottom up. With the identity link option, the horizontal axis is the model output. In Figure 31, the fixed gray vertical line represents the base value, and movement to the right or left indicates increasing or decreasing support for the class shown. The colored path illustrates how the selected features contribute to shift the output toward the “Attack” class. The overall effect of these contributions led to an output of f(x)=0.71, which supports the “Attack” class for this instance.

**Figure 32:**
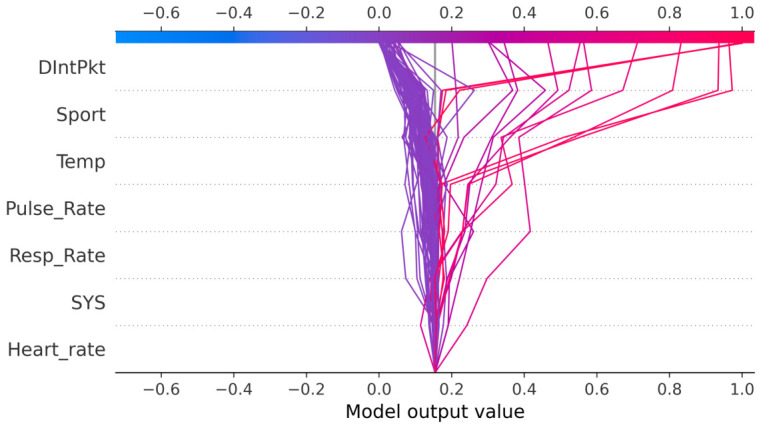
SHAP decision plot for 100 instances—WUSTL-EHMS.

Compared with Figure 31, which shows a single instance, Figure 32 shows the cumulative decision paths for 100 instances. Each colored path represents one observation and gives a more comprehensive picture of how cumulative feature contributions differ across multiple instances for the “Attack” class.

## 7. Discussion

This section discusses the main findings of the study by comparing the baseline and proposed methods across the three datasets. It also examines the effect of leakage-prone features, computational efficiency, parameter sensitivity and robustness, representative FS baselines, and comparisons with related studies.

On the IoTID20 dataset, AHGA-SA maintained the baseline’s high detection performance while substantially enhancing the model’s compactness and efficiency. As shown in Table 17, reducing the feature set from 55 to 12 corresponds to a 78.18% relative reduction, with slight improvements in accuracy, precision, specificity, and AUC-ROC. At the same time, MCC, recall, F1 score, and FNR were preserved while FPR was reduced, indicating a slight decrease in false alarms. In addition, the proposed approach enhanced computational efficiency, with a 67.52% reduction in training time, a 49.49% reduction in testing time, a 3.92% reduction in training CPU usage, a 3.30% reduction in testing CPU usage, a 69.22% reduction in training RSS overhead, and a 99.58% reduction in testing RSS overhead.

On the WUSTL-EHMS dataset, AHGA-SA improved the detection performance while substantially reducing the model complexity, as shown in Table 18. The number of features decreased by 76.67%. At the same time, accuracy, MCC, recall, F1 score, and AUC-ROC improved, but precision and specificity slightly decreased and FPR slightly increased. Notably, FNR decreased by 30.91%, which is important for reducing missed attacks. Computational efficiency also improved, with training time reduced by 49.30%, testing time by 51.26%, training CPU usage by 4.24%, testing CPU usage by 4.14%, training RSS overhead by 98.99%, and testing RSS overhead by 80.00%.

For the Edge-IIoTset (ML) dataset, AHGA-SA substantially improved model compactness while maintaining high detection performance, as shown in Table 19. The number of features decreased by 72.73%. At the same time, accuracy, precision, recall, specificity, and F1 score slightly increased, while MCC remained the same. Moreover, both FPR and FNR were reduced, indicating small reductions in false alarms and missed attacks. While AUC-ROC slightly decreased, the proposed method improved computational efficiency, with a 61.53% reduction in training time, a 38.07% reduction in testing time, a 5.64% reduction in training CPU usage, a 3.12% reduction in testing CPU usage, an 83.15% reduction in training RSS overhead, and a 98.91% reduction in testing RSS overhead.

**Figure 33:**
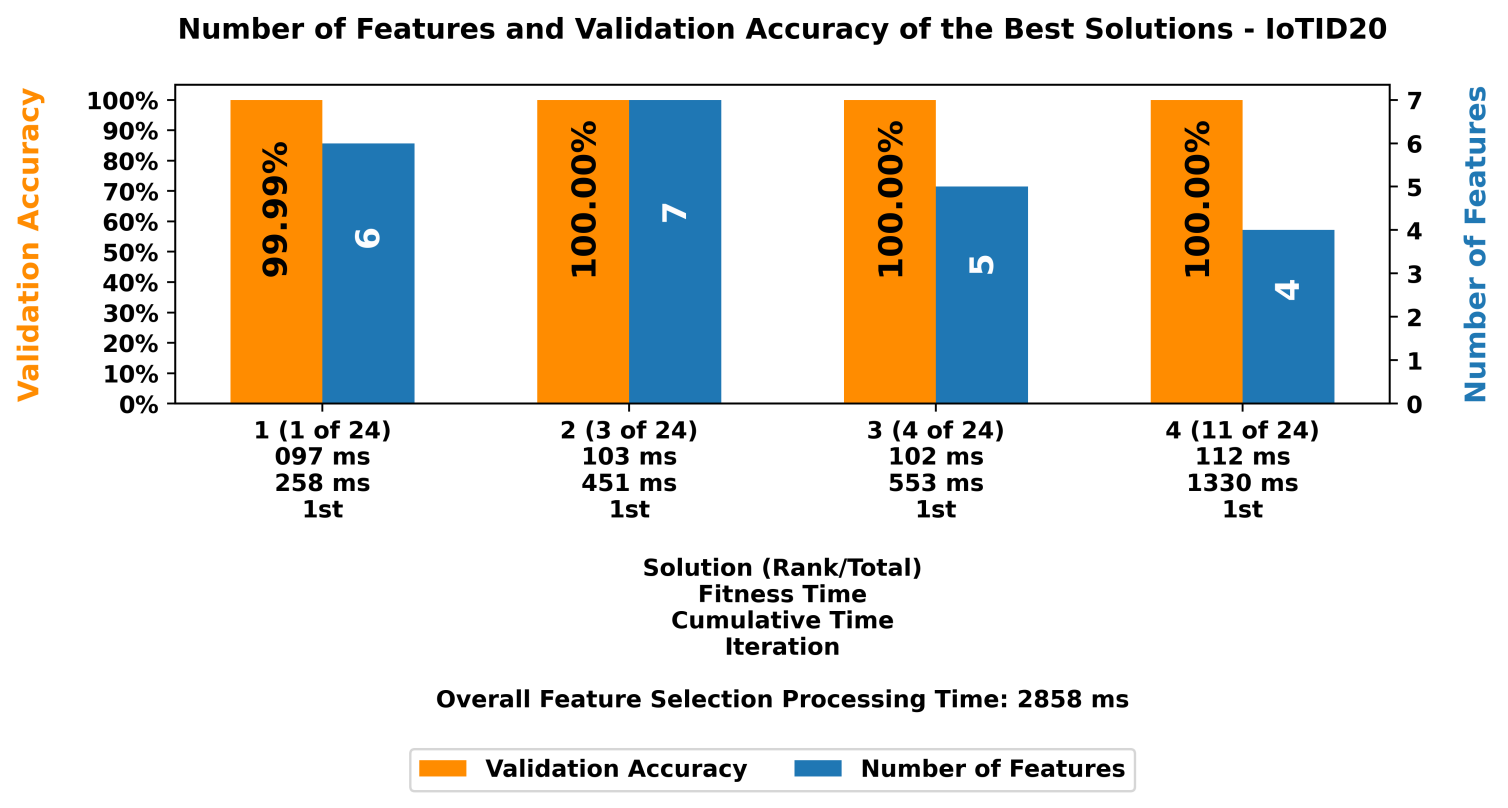
Diagnostic AHGA-SA solutions before feature removal—IoTID20.

To assess the effect of retaining leakage-prone fields, a diagnostic analysis was conducted before finalizing feature removal. As shown in Table 20, AHGA-SA selected extremely small subsets with perfect accuracy when such fields were retained, suggesting inflated FS behavior rather than reliable generalization. Figure 33 further illustrates this behavior for the representative IoTID20 case.

In terms of computational cost, the population size is a key factor in population-based optimization algorithms, such as GAs. A larger population may increase search diversity and potentially improve the chance of finding high-quality feature subsets, but it also increases the number of evaluated candidate solutions and the overall FS runtime [74]. Since AHGA-SA uses a fixed two-stage search design, the effect of population size was further examined on the representative IoTID20 dataset while keeping the remaining settings unchanged. To isolate the effect of population size on the number of evaluated candidate solutions and runtime, this comparison was conducted without duplicate removal only for this sensitivity check. As shown in Table 21, increasing the population size from four to eight doubled the evaluated candidate solutions from 24 to 48 and increased the FS time from ∼3319 ms to ∼7104 ms. However, the classification accuracy did not improve, decreasing slightly from 99.04% to 99.03%, although the selected subset size was reduced from 12 to 10 features. Therefore, the adopted population size of four provides a more practical accuracy–compactness–cost trade-off for the lightweight AHGA-SA setting.

To further examine whether the compact AHGA-SA configuration produced stable outcomes under stochastic search behavior, a repeated-seed robustness check was conducted using the full adopted AHGA-SA setting on IoTID20, including the duplicate-removal strategy. In this check, the data split and all AHGA-SA parameters were kept fixed, while only the random seed controlling the stochastic search operations was changed across five runs. As shown in Table 22, AHGA-SA maintained highly consistent classification accuracy, compact feature subsets, and comparable FS runtime across different random seeds. The accuracy ranged from 98.99% to 99.04%, while the number of selected features ranged from 11 to 14. This indicates that the observed performance and compactness were not dependent on a single favorable random run.

The XGBoost preselection depth was also examined on the same representative IoTID20 dataset because this step determines the initial reduced feature space passed to AHGA-SA. As shown in Table 23, increasing the number of XGBoost estimators from two to five and ten increased the number of retained preselected features from 31 to 39 and 41, respectively, and also increased the preselection time from 137 ms to 166 ms and 194 ms. Since the purpose of this step is to provide a lightweight initial feature ranking rather than a final classifier, two estimators were retained as a practical setting that reduces the search space and preselection cost before the AHGA-SA search.

A representative FS baseline comparison was conducted on IoTID20 to contextualize the proposed AHGA-SA framework against commonly used standalone FS alternatives. All methods were evaluated using the same data split, the same final XGBoost classifier, and the same hardware/software setup. The baseline FS methods were used independently, and each method selected features according to its own selection mechanism. RF importance used RandomForestClassifier with random_state=42 and retained features with importance scores greater than zero. MI used mutual_info_classif with random_state=42 and retained features with MI scores above the mean MI score. L1-based selection used L1-regularized logistic regression and retained features with non-zero coefficients. GA-based FS used the same GA-related parameters as AHGA-SA, namely N=4, $C_{r}=0.7$, $M_{r}=0.2$, and tournament size S=2, with an initial population followed by five generations, resulting in 24 evaluated candidate solutions. PSO-based FS used four particles, six iterations, inertia weight w=0.7, cognitive coefficient $c_{1}=1.5$, and social coefficient $c_{2}=1.5$, resulting in 24 evaluated candidate solutions. SA-based FS used the same annealing-related parameters as AHGA-SA, namely, initial temperature T=95 and cooling rate α=0.90, with one initial solution followed by 23 neighbor evaluations, resulting in 24 evaluated candidate solutions. This setup allowed the comparison to reflect the quality of the selected feature subsets in terms of detection accuracy, subset compactness, and FS cost. Table 24 summarizes the results.

The results show that MI achieved the highest accuracy, with a marginal gain of 0.01% over AHGA-SA. However, this required 32 selected features and substantially higher FS time. In contrast, AHGA-SA achieved 99.04% accuracy using only 12 features and the lowest FS time among the compared methods. Compared with GA-based, PSO-based, and SA-based FS, AHGA-SA selected fewer features while maintaining higher detection accuracy and lower FS time. These results show that AHGA-SA provides a favorable accuracy–compactness–cost trade-off rather than only optimizing detection accuracy. It is worth noting that the additional representative analyses reported above were conducted on IoTID20 because it is the largest dataset in this study and represents the most computationally demanding setting.

In the adopted configuration, a tournament size of two was chosen to preserve search diversity and maintain sufficient selection pressure toward fitter individuals. Notably, a low crossover rate may reduce the creation of new candidate solutions and lead to premature convergence, while a high mutation rate may disrupt high-quality solutions and impede convergence. Therefore, a crossover rate of 70% and a mutation rate of 20% were chosen to maintain diversity and support convergence stability. For SA, the initial temperature was set to 95 and the cooling rate to 90% to support broader exploration in the early part of the annealing process, followed by greater exploitation as the temperature decreases.

To further examine the stability of the GA and SA parameter settings, a targeted representative fixed-seed parameter-robustness analysis was conducted on IoTID20, as reported in Appendix B. In that analysis, one GA/SA parameter was varied at a time, while the data split, XGBoost settings, hardware/software setup, and evaluation protocol were kept unchanged. FS time is reported as the mean and standard deviation over five consecutive measured runs. The selected subset size and accuracy showed no observed variation across the measured runs for each configuration.

Beyond parameter-setting robustness, the stochastic nature of the GA-based search process remains a relevant methodological consideration. In particular, population initialization introduces randomness that helps maintain search diversity, but it may also influence population coverage, convergence behavior, and the quality of the final selected feature subset [75]. This issue can be mitigated through more structured initialization strategies, such as low-discrepancy sequences, Latin Hypercube sampling, probability-distribution-based initialization, chaotic initialization, and hybrid initialization schemes, which have been investigated to improve search-space coverage, population diversity, and optimization performance [76,77].

To better contextualize the proposed framework, Table 25 compares AHGA-SA with other studies on the three datasets. AHGA-SA achieved the highest reported accuracy among the compared studies on the three datasets while using the smallest feature subsets. These results suggest the effectiveness of AHGA-SA in obtaining a competitive accuracy–compactness trade-off across datasets with different characteristics.

## 8. Conclusions and Future Work

This paper presented AHGA-SA, an adaptive hybrid approach to FS for intrusion detection in IoT-oriented environments. The framework used GA-based exploration, SA-based exploitation, and XGBoost-based feature preselection, along with a duplicate-removal strategy, multi-point fitness evaluation, and a two-stage AHGA-SA search design, to discover compact, informative feature subsets while maintaining or enhancing detection performance at an acceptable computational cost.

AHGA-SA was tested on three recent benchmark datasets from key IoT-oriented environments, including IoT, IoMT, and IIoT. The findings showed that detection performance was maintained or improved while the number of selected features was reduced, with accuracies of 99.04% on IoTID20 (using 12 features), 98.25% on WUSTL-EHMS (using seven features), and 99.18% on Edge-IIoTset (ML) (using nine features). The results also showed lower training and testing times, CPU usage, and RSS overhead compared to the baseline. The selected feature subsets and the FS process were reported, and SHAP-based XAI was used to explain the contribution of the selected features to the IDS decision-making process.

One limitation is the stochastic nature of the GA-based search process, particularly population initialization, which may lead to variability in the selected feature subsets. Future research may investigate more controlled population-generation mechanisms to better balance population diversity and solution quality, reduce reliance on purely random initialization, and improve the consistency of AHGA-SA across multiple experiments. Another future direction is to apply AHGA-SA to additional datasets from different IoT-oriented domains to further evaluate its robustness and generalizability.

## Figures and Tables

**Table 1:** Dataset-based summary of IDS studies on IoTID20, WUSTL-EHMS, and Edge-IIoTset (ML).

Dataset	Ref.	Year	FS Method(s)	No. of Feat.	Acc.	Limitations
IoTID20	[45]	2021	PSO	20	98.20%	Single-dataset evaluation only; limited experimental setup reporting; no resource-cost reporting.
	[46]	2023	PCC	15	99.00%	No selected feature subset reporting; limited experimental setup reporting; no runtime reporting (FS/training/testing); no resource-cost reporting.
	[47]	2025	BBOA	40	98.87%	No selected feature subset reporting; limited experimental setup reporting; no runtime reporting (FS/training/testing); no resource-cost reporting.
	[48]	2026	FA	47	98.20%	No selected feature subset reporting; reliance on data-level balancing/resampling.
WUSTL-EHMS	[49]	2022	PSO	8	96.00%	Single-dataset evaluation only; no selected feature subset reporting; limited experimental setup reporting; no runtime reporting (FS/training/testing); no resource-cost reporting.
	[50]	2023	LRGU-MIFS	25	96.70%	Single-dataset evaluation only; no selected feature subset reporting; limited experimental setup reporting; no runtime reporting (FS/training/testing); no resource-cost reporting.
	[51]	2024	RFE-DT	8	97.85%	Single-dataset evaluation only; no selected feature subset reporting; limited experimental setup reporting; no runtime reporting (FS/training/testing); limited resource-cost reporting.
	[52]	2025	RSFS + CFS	18	96.37%	No selected feature subset reporting; no resource-cost reporting.
Edge-IIoTset (ML)	[53]	2022	SMOTE + PCC + variance threshold	15	98.43%	Single-dataset evaluation only; no selected feature subset reporting; limited experimental setup reporting; no runtime reporting (FS/training/testing); no resource-cost reporting; reliance on data-level balancing/resampling.
	[54]	2023	Fisher-score ranking	45	94.54%	Single-dataset evaluation only; no selected feature subset reporting; limited experimental setup reporting; no runtime reporting (FS/training/testing); no resource-cost reporting.
	[55]	2023	ROC-value feature importance + RF	15	99.16%	No selected feature subset reporting; limited experimental setup reporting; no runtime reporting (FS/training/testing); no resource-cost reporting; reliance on data-level balancing/resampling.
	[56]	2025	Ensemble (7 algorithms)	34	97.64%	Single-dataset evaluation only; no selected feature subset reporting; limited experimental setup reporting; limited resource-cost reporting.

Ref.: Reference; No. of Feat.: Number of Features.

**Table 2:** Summary of feature statistics for each dataset.

Dataset	Initial	Removed	Final
IoTID20	83	18	65
WUSTL-EHMS	43	7	36
Edge-IIoTset (ML)	61	19	42

**Table 3:** Common performance metrics and their formulas.

Measure	Formula
Acc	$\frac{TP\;+\;TN}{TP\;+\;TN\;+\;FP\;+\;FN}$
MCC	$\frac{TP\;\cdot \;TN\;-\;FP\;\cdot \;FN}{\sqrt{\;(TP\;+\;FP)\;(TP\;+\;FN)\;(TN\;+\;FP)\;(TN\;+\;FN)}}$
Precision	$\mathrm{PPV}=\frac{TP}{TP\;+\;FP}$
Recall	$\mathrm{TPR}=\frac{TP}{TP\;+\;FN}$
Specificity	$\mathrm{TNR}=\frac{TN}{TN\;+\;FP}$
F1 score	$2\times \frac{\mathrm{PPV}\;\times \;\mathrm{TPR}}{\mathrm{PPV}\;+\;\mathrm{TPR}}$
FPR	1−Specificity
FNR	1−Recall

**Table 4:** Hardware and software setup.

Component	Specification (Version)
CPU	Intel Core i9-14900K 3.20 GHz
RAM	192 GB DDR5 5400 MT/s
Storage	Samsung 990 PRO NVMe SSD 2TB
Operating System	Windows 11 Pro (25H2)
Code Editor	Visual Studio Code (1.112)
Programming Language	Python (3.14.3)
Main Libraries	NumPy, pandas, scikit-learn, SHAP, XGBoost, psutil

**Table 5:** Algorithm parameters and programming settings.

Component	Specification
Baseline FS	RF Classifier (random_state = 42)
Baseline Detection Model	XGBoost Classifier (n_estimators = *)
XGBoost Feature Preselection	XGBoost Classifier (n_estimators = 2)
AHGA-SA Fitness Classifier	XGBoost Classifier (n_estimators = *)
Final Detection Model	XGBoost Classifier (n_estimators = *)
XGBoost Evaluation Metric	logloss
XGBoost Random State	42
Train/Test Split	80%/20%; shuffled; random_state = 10
Internal Train/Validation Split	80%/20% from the training set; shuffled; stratified; random_state = 10
Population Size (*N*)	4
Fixed AHGA-SA Search Stages ($S_{\max}$)	2
Crossover Rate ($C_{r}$)	0.7
Mutation Rate ($M_{r}$)	0.2
Tournament Size (*S*)	2
Initial Temperature (*T*)	95
Cooling Rate (α)	0.9

* n_estimators: 25 for IoTID20, 150 for WUSTL-EHMS, and 75 for Edge-IIoTset (ML).

**Table 6:** Feature importance computed using RF—IoTID20.

No.	Feature	Imp. (%)	No.	Feature	Imp. (%)	No.	Feature	Imp. (%)
1	Flow_Duration	13.589	20	Fwd_Header_Len	1.605	39	SYN_Flag_Cnt	0.421
2	Init_Bwd_Win_Byts	12.838	21	Fwd_Pkt_Len_Max	1.520	40	Fwd_IAT_Std	0.343
3	Flow_Pkts/s	6.097	22	TotLen_Bwd_Pkts	1.488	41	Fwd_IAT_Min	0.294
4	Flow_IAT_Max	5.138	23	Pkt_Len_Min	1.425	42	Tot_Bwd_Pkts	0.273
5	ACK_Flag_Cnt	4.384	24	Flow_Byts/s	1.423	43	Tot_Fwd_Pkts	0.222
6	Bwd_Pkts/s	3.423	25	Fwd_IAT_Tot	1.233	44	Bwd_Pkt_Len_Std	0.137
7	Bwd_Header_Len	3.323	26	Bwd_Pkt_Len_Min	1.201	45	Down/Up_Ratio	0.117
8	Flow_IAT_Min	3.069	27	Pkt_Len_Mean	1.150	46	Active_Min	0.036
9	Flow_IAT_Mean	2.842	28	TotLen_Fwd_Pkts	1.109	47	Bwd_PSH_Flags	0.035
10	Idle_Mean	2.724	29	Fwd_Pkt_Len_Mean	1.072	48	Active_Mean	0.026
11	Idle_Min	2.675	30	Fwd_Pkts/s	1.069	49	FIN_Flag_Cnt	0.009
12	Idle_Std	2.613	31	Protocol	1.045	50	CWE_Flag_Count	0.001
13	Fwd_Pkt_Len_Std	2.599	32	Fwd_Pkt_Len_Min	1.010	51	RST_Flag_Cnt	0.001
14	Flow_IAT_Std	2.304	33	Fwd_IAT_Mean	0.888	52	ECE_Flag_Cnt	0.001
15	Pkt_Len_Max	2.194	34	Bwd_Pkt_Len_Mean	0.814	53	Active_Std	0.0002
16	Bwd_IAT_Tot	2.035	35	Bwd_IAT_Std	0.665	54	Bwd_URG_Flags	0.00001
17	Bwd_IAT_Mean	2.008	36	Pkt_Len_Var	0.613	55	URG_Flag_Cnt	0.00001
18	Bwd_IAT_Min	1.947	37	Fwd_Act_Data_Pkts	0.580			
19	Bwd_Pkt_Len_Max	1.938	38	Pkt_Len_Std	0.432			

Imp.: importance.

**Table 7:** Performance metrics for the baseline model—IoTID20.

Metric	Score	Metric	Score
Acc	99.03%	MCC	0.92
Precision	99.05%	Recall	99.93%
Specificity	85.72%	F1 score	99.49%
FPR	14.28%	FNR	0.07%
AUC-ROC	99.50%	No. of features	55
Normal samples	7905	Attack samples	117,252
Training time	334.61 ms	Testing time	8.89 ms
Training CPU usage	84.37%	Testing CPU usage	80.29%
Training RSS overhead	88.24 MB	Testing RSS overhead	2.40 MB

**Table 8:** Feature importance from XGBoost preselection—IoTID20.

No.	Feature	Imp. (%)	No.	Feature	Imp. (%)
1	ACK_Flag_Cnt	85.743	17	Fwd_Pkt_Len_Max	0.036
2	Fwd_Pkt_Len_Std	6.125	18	Bwd_IAT_Tot	0.033
3	Flow_Duration	2.827	19	TotLen_Fwd_Pkts	0.026
4	Protocol	1.149	20	Idle_Mean	0.022
5	Fwd_Pkt_Len_Min	1.045	21	Bwd_IAT_Mean	0.013
6	Init_Bwd_Win_Byts	0.998	22	Bwd_Pkts/s	0.012
7	Flow_IAT_Max	0.536	23	Fwd_IAT_Tot	0.012
8	Bwd_Pkt_Len_Min	0.341	24	Bwd_Pkt_Len_Mean	0.009
9	Bwd_Header_Len	0.253	25	Fwd_IAT_Mean	0.005
10	Bwd_IAT_Std	0.208	26	Flow_IAT_Mean	0.005
11	Flow_Pkts/s	0.186	27	Flow_Byts/s	0.003
12	Bwd_Pkt_Len_Max	0.138	28	Tot_Fwd_Pkts	0.002
13	Pkt_Len_Mean	0.104	29	Bwd_Pkt_Len_Std	0.001
14	Pkt_Len_Min	0.074	30	Bwd_IAT_Min	0.001
15	Fwd_Pkts/s	0.048	31	Flow_IAT_Min	0.001
16	Flow_IAT_Std	0.043			

Imp.: importance.

**Table 9:** Selected feature subsets of the best solutions—IoTID20.

No.	No. of Feat.	Selected Features
1	8	ACK_Flag_Cnt, Fwd_Pkt_Len_Std, Protocol, Flow_IAT_Max, Pkt_Len_Min, Idle_Mean, Fwd_IAT_Mean, Bwd_Pkt_Len_Min
2	12	ACK_Flag_Cnt, Fwd_Pkt_Len_Std, Fwd_Pkt_Len_Min, Flow_Pkts/s, Bwd_Header_Len, Pkt_Len_Min, Flow_IAT_Std, Idle_Mean, Bwd_IAT_Std, Fwd_IAT_Mean, Bwd_Pkt_Len_Min, Bwd_IAT_Min
3	10	ACK_Flag_Cnt, Fwd_Pkt_Len_Std, Init_Bwd_Win_Byts, Bwd_Header_Len, Pkt_Len_Min, Idle_Mean, Bwd_IAT_Std, Fwd_IAT_Mean, Bwd_Pkt_Len_Min, Bwd_IAT_Min
4	8	Init_Bwd_Win_Byts, Fwd_Pkt_Len_Min, Protocol, Flow_Pkts/s, Bwd_Header_Len, Pkt_Len_Min, Fwd_IAT_Mean, Bwd_Pkt_Len_Min
5	8	Init_Bwd_Win_Byts, Fwd_Pkt_Len_Min, Protocol, Flow_Pkts/s, Bwd_Header_Len, Pkt_Len_Min, Idle_Mean, Bwd_Pkt_Len_Min
6	12	ACK_Flag_Cnt, Fwd_Pkt_Len_Std, Flow_Duration, Init_Bwd_Win_Byts, Protocol, Flow_IAT_Max, Flow_Pkts/s, Pkt_Len_Min, TotLen_Fwd_Pkts, Bwd_IAT_Std, Bwd_Pkt_Len_Min, Bwd_IAT_Min
7	12	ACK_Flag_Cnt, Fwd_Pkt_Len_Std, Flow_Duration, Init_Bwd_Win_Byts, Protocol, Flow_IAT_Max, Bwd_Header_Len, TotLen_Fwd_Pkts, Idle_Mean, Bwd_IAT_Std, Bwd_Pkt_Len_Min, Bwd_IAT_Min

No. of Feat.: number of features.

**Table 10:** Performance Metrics for the Final Model—IoTID20.

Metric	Score	Metric	Score
Acc	99.04%	MCC	0.92
Precision	99.06%	Recall	99.93%
Specificity	85.87%	F1 score	99.49%
FPR	14.13%	FNR	0.07%
AUC-ROC	99.54%	No. of features	12
Normal samples	7905	Attack samples	117,252
Training time	108.67 ms	Testing time	4.49 ms
Training CPU usage	81.06%	Testing CPU usage	77.64%
Training RSS overhead	27.16 MB	Testing RSS overhead	0.01 MB

**Table 11:** Performance metrics for the baseline model—WUSTL-EHMS.

Metric	Score	Metric	Score
Acc	97.92%	MCC	0.89
Precision	95.96%	Recall	84.89%
Specificity	99.55%	F1 score	90.09%
FPR	0.45%	FNR	15.11%
AUC-ROC	99.29%	No. of features	30
Normal samples	2900	Attack samples	364
Training time	136.56 ms	Testing time	2.77 ms
Training CPU usage	75.41%	Testing CPU usage	71.95%
Training RSS overhead	5.94 MB	Testing RSS overhead	0.05 MB

**Table 12:** XGBoost preselection feature importance—WUSTL-EHMS.

No.	Feature	Imp. (%)	No.	Feature	Imp. (%)
1	SrcLoad	71.196	8	ST	0.808
2	DIntPkt	10.037	9	SpO2	0.802
3	SrcJitter	6.155	10	DstJitter	0.502
4	Resp_Rate	2.768	11	Temp	0.435
5	DstLoad	2.373	12	SYS	0.391
6	Sport	2.339	13	Heart_rate	0.056
7	Pulse_Rate	2.138			

Imp.: importance.

**Table 13:** Performance metrics for the final model—WUSTL-EHMS.

Metric	Score	Metric	Score
Acc	98.25%	MCC	0.91
Precision	94.49%	Recall	89.56%
Specificity	99.34%	F1 score	91.96%
FPR	0.66%	FNR	10.44%
AUC-ROC	99.41%	No. of features	7
Normal samples	2900	Attack samples	364
Training time	69.23 ms	Testing time	1.35 ms
Training CPU usage	72.21%	Testing CPU usage	68.97%
Training RSS overhead	0.06 MB	Testing RSS overhead	0.01 MB

**Table 14:** Performance metrics for the baseline model—Edge-IIoTset (ML).

Metric	Score	Metric	Score
Acc	99.16%	MCC	0.97
Precision	99.57%	Recall	99.43%
Specificity	97.64%	F1 score	99.50%
FPR	2.36%	FNR	0.57%
AUC-ROC	99.97%	No. of features	33
Normal samples	4830	Attack samples	26,730
Training time	215.18 ms	Testing time	4.86 ms
Training CPU usage	83.74%	Testing CPU usage	78.10%
Training RSS overhead	22.85 MB	Testing RSS overhead	0.92 MB

**Table 15:** XGBoost preselection feature importance—Edge-IIoTset (ML).

No.	Feature	Imp. (%)	No.	Feature	Imp. (%)
1	mqtt.hdrflags	25.030	7	tcp.ack	5.849
2	tcp.len	22.665	8	tcp.ack_raw	3.191
3	tcp.seq	12.579	9	udp.time_delta	0.472
4	http.request.version	11.081	10	tcp.checksum	0.325
5	tcp.flags	10.308	11	arp.opcode	0.011
6	tcp.connection.synack	8.490			

Imp.: importance.

**Table 16:** Performance metrics for the final model—Edge-IIoTset (ML).

Metric	Score	Metric	Score
Acc	99.18%	MCC	0.97
Precision	99.58%	Recall	99.45%
Specificity	97.68%	F1 score	99.52%
FPR	2.32%	FNR	0.55%
AUC-ROC	99.92%	No. of features	9
Normal samples	4830	Attack samples	26,730
Training time	82.77 ms	Testing time	3.01 ms
Training CPU usage	79.02%	Testing CPU usage	75.66%
Training RSS overhead	3.85 MB	Testing RSS overhead	0.01 MB

**Table 17:** Comparison of baseline and proposed methods—IoTID20.

Metric	Baseline	Proposed	Difference	Direction
Acc	99.03%	99.04%	+0.01%	Positive
MCC	0.92	0.92	0.00	No change
Precision	99.05%	99.06%	+0.01%	Positive
Recall	99.93%	99.93%	0.00%	No change
Specificity	85.72%	85.87%	+0.15%	Positive
F1 score	99.49%	99.49%	0.00%	No change
FPR	14.28%	14.13%	−0.15%	Positive
FNR	0.07%	0.07%	0.00%	No change
AUC-ROC	99.50%	99.54%	+0.04%	Positive
No. of features	55	12	−43	Positive
Training time	334.61 ms	108.67 ms	−225.94 ms	Positive
Testing time	8.89 ms	4.49 ms	−4.40 ms	Positive
Training CPU usage	84.37%	81.06%	−3.31%	Positive
Testing CPU usage	80.29%	77.64%	−2.65%	Positive
Training RSS overhead	88.24 MB	27.16 MB	−61.08 MB	Positive
Testing RSS overhead	2.40 MB	0.01 MB	−2.39 MB	Positive

**Table 18:** Comparison of baseline and proposed methods—WUSTL-EHMS.

Metric	Baseline	Proposed	Difference	Direction
Acc	97.92%	98.25%	+0.33%	Positive
MCC	0.89	0.91	+0.02	Positive
Precision	95.96%	94.49%	−1.47%	Negative
Recall	84.89%	89.56%	+4.67%	Positive
Specificity	99.55%	99.34%	−0.21%	Negative
F1 score	90.09%	91.96%	+1.87%	Positive
FPR	0.45%	0.66%	+0.21%	Negative
FNR	15.11%	10.44%	−4.67%	Positive
AUC-ROC	99.29%	99.41%	+0.12%	Positive
No. of features	30	7	−23	Positive
Training time	136.56 ms	69.23 ms	−67.33 ms	Positive
Testing time	2.77 ms	1.35 ms	−1.42 ms	Positive
Training CPU usage	75.41%	72.21%	−3.20%	Positive
Testing CPU usage	71.95%	68.97%	−2.98%	Positive
Training RSS overhead	5.94 MB	0.06 MB	−5.88 MB	Positive
Testing RSS overhead	0.05 MB	0.01 MB	−0.04 MB	Positive

**Table 19:** Comparison of baseline and proposed methods—Edge-IIoTset (ML).

Metric	Baseline	Proposed	Difference	Direction
Acc	99.16%	99.18%	+0.02%	Positive
MCC	0.97	0.97	0.00	No Change
Precision	99.57%	99.58%	+0.01%	Positive
Recall	99.43%	99.45%	+0.02%	Positive
Specificity	97.64%	97.68%	+0.04%	Positive
F1 score	99.50%	99.52%	+0.02%	Positive
FPR	2.36%	2.32%	−0.04%	Positive
FNR	0.57%	0.55%	−0.02%	Positive
AUC-ROC	99.97%	99.92%	−0.05%	Negative
No. of features	33	9	−24	Positive
Training time	215.18 ms	82.77 ms	−132.41 ms	Positive
Testing time	4.86 ms	3.01 ms	−1.85 ms	Positive
Training CPU usage	83.74%	79.02%	−4.72%	Positive
Testing CPU usage	78.10%	75.66%	−2.44%	Positive
Training RSS overhead	22.85 MB	3.85 MB	−19.00 MB	Positive
Testing RSS overhead	0.92 MB	0.01 MB	−0.91 MB	Positive

**Table 20:** Diagnostic effect of retaining leakage-prone features across datasets.

Dataset	No. of Feat.	Acc.	Selected Features
IoTID20	4	100.00%	Dst_Port, Flow_Duration, Timestamp, Init_Bwd_Win_Byts
WUSTL-EHMS	1	100.00%	SrcMac
Edge-IIoTset (ML)	2	100.00%	dns.qry.name.len, udp.port

**Table 21:** Population-size sensitivity analysis for AHGA-SA on IoTID20.

Population Size	Evaluated Solutions	FS Time	No. of Features	Acc.
4	24	∼3319 ms	12	99.04%
8	48	∼7104 ms	10	99.03%

**Table 22:** Repeated-seed robustness check for AHGA-SA on IoTID20.

Metric	Mean	Std.	Min	Max
FS time (ms)	3591	309	3137	3891
No. of features	12.80	1.30	11	14
Acc.	99.02%	0.02%	98.99%	99.04%

**Table 23:** XGBoost preselection-depth check on IoTID20.

XGBoost Estimators	Preselected Features	Preselection Time
2	31	137 ms
5	39	166 ms
10	41	194 ms

**Table 24:** Representative FS baseline comparison on IoTID20.

FS Method	FS Time	No. of Features	Acc.
RF importance	34,702 ms	55	99.03%
Mutual information	85,590 ms	32	99.05%
L1-based selection	84,916 ms	55	99.03%
GA-based FS	5268 ms	33	99.03%
PSO-based FS	5274 ms	38	99.02%
SA-based FS	4872 ms	33	98.80%
AHGA-SA	3166 ms	12	99.04%

**Table 25:** Comparison of the proposed method with related work across the three datasets.

Dataset	Study	FS Method	No. of Features	Classifier	Acc.
IoTID20	[45]	PSO	20	LSTM	98.20%
	[46]	PCC	15	PCC-CNN	99.00%
	[47]	BBOA	40	LSTM	98.87%
	[48]	FA	47	FA-CNN-RNN	98.20%
	This study	AHGA-SA	12	XGBoost	99.04%
WUSTL-EHMS	[49]	PSO	8	DNN	96.00%
	[50]	LRGU-MIFS	25	FST-LSTM	96.70%
	[51]	RFE-DT	8	DT	97.85%
	[52]	RSFS + CFS	18	DNN	96.37%
	This study	AHGA-SA	7	XGBoost	98.25%
Edge-IIoTset (ML)	[53]	SMOTE + PCC + variance threshold	15	DT	98.43%
	[54]	Fisher-score ranking	45	RF	94.54%
	[55]	ROC-value feature importance + RF	15	RF	99.16%
	[56]	Ensemble (7 algorithms)	34	XGBoost	97.64%
	This study	AHGA-SA	9	XGBoost	99.18%

## Data Availability

The datasets used in this study are publicly available from their original sources: IoTID20 [57], WUSTL-EHMS [60], and Edge-IIoTset [61]. No new datasets were generated.

## Appendix A. Repeated Operational-Measurement Variability

**Table A1:** Repeated operational-measurement variability across datasets.

Dataset	Model	Metric	Mean	Std.
IoTID20	Baseline	Training time	334.61 ms	±21.97 ms
		Testing time	8.89 ms	±1.43 ms
		Training CPU usage	84.37%	±1.46%
		Testing CPU usage	80.29%	±2.08%
		Training RSS overhead	88.24 MB	±7.66 MB
		Testing RSS overhead	2.40 MB	±0.23 MB
	Proposed	Training time	108.67 ms	±9.84 ms
		Testing time	4.49 ms	±0.35 ms
		Training CPU usage	81.06%	±1.83%
		Testing CPU usage	77.64%	±2.15%
		Training RSS overhead	27.16 MB	±6.50 MB
		Testing RSS overhead	0.01 MB	±0.01 MB
WUSTL-EHMS	Baseline	Training time	136.56 ms	±10.92 ms
		Testing time	2.77 ms	±0.15 ms
		Training CPU usage	75.41%	±2.33%
		Testing CPU usage	71.95%	±2.89%
		Training RSS overhead	5.94 MB	±0.35 MB
		Testing RSS overhead	0.05 MB	±0.02 MB
	Proposed	Training time	69.23 ms	±4.92 ms
		Testing time	1.35 ms	±0.07 ms
		Training CPU usage	72.21%	±1.52%
		Testing CPU usage	68.97%	±2.03%
		Training RSS overhead	0.06 MB	±0.02 MB
		Testing RSS overhead	0.01 MB	±0.01 MB
Edge-IIoTset (ML)	Baseline	Training time	215.18 ms	±16.58 ms
		Testing time	4.86 ms	±0.79 ms
		Training CPU usage	83.74%	±1.65%
		Testing CPU usage	78.10%	±1.81%
		Training RSS overhead	22.85 MB	±3.16 MB
		Testing RSS overhead	0.92 MB	±0.10 MB
	Proposed	Training time	82.77 ms	±6.97 ms
		Testing time	3.01 ms	±0.20 ms
		Training CPU usage	79.02%	±1.36%
		Testing CPU usage	75.66%	±2.13%
		Training RSS overhead	3.85 MB	±0.31 MB
		Testing RSS overhead	0.01 MB	±0.01 MB

Std.: standard deviation over five measured runs.

## Appendix B. Targeted Parameter-Robustness Analysis

**Table A2:** Targeted AHGA-SA parameter-robustness analysis on IoTID20.

Case	Setting	Parameter Change	No. of Features	Acc.	FS Time Mean	FS Time Std.
1	Default	$C_{r}=0.7$, $M_{r}=0.2$, T=95, α=0.90	11	99.04%	2984 ms	±35 ms
2	Crossover low	$C_{r}=0.6$	11	99.04%	2976 ms	±34 ms
3	Crossover high	$C_{r}=0.8$	11	99.04%	3042 ms	±41 ms
4	Mutation low	$M_{r}=0.1$	11	99.04%	2969 ms	±33 ms
5	Mutation high	$M_{r}=0.3$	11	99.04%	2995 ms	±31 ms
6	Temperature low	T=70	11	99.04%	3058 ms	±42 ms
7	Temperature high	T=120	11	99.04%	3053 ms	±48 ms
8	Cooling low	α=0.85	11	99.04%	3044 ms	±39 ms
9	Cooling high	α=0.95	11	99.04%	3067 ms	±56 ms

Std.: standard deviation over five measured runs.
